# Scrutinizing Domains of Executive Function in Binge Eating Disorder: A Systematic Review and Meta-Analysis

**DOI:** 10.3389/fpsyt.2020.00288

**Published:** 2020-04-17

**Authors:** Maria Elisa Gisbert Cury, Arthur Berberian, Bruno Sini Scarpato, Jess Kerr-Gaffney, Flavia H. Santos, Angélica Medeiros Claudino

**Affiliations:** ^1^Eating Disorders Program (PROATA), Department of Psychiatry, Universidade Federal de São Paulo (UNIFESP), São Paulo, Brazil; ^2^Interdisciplinary Laboratory of Clinical Neurosciences (LiNC), Department of Psychiatry, Universidade Federal de São Paulo (UNIFESP), São Paulo, Brazil; ^3^Department of Psychiatry, Universidade Federal de São Paulo (UNIFESP), São Paulo, Brazil; ^4^Department of Psychological Medicine, Institute of Psychiatry, Psychology, and Neuroscience, King's College London, London, United Kingdom; ^5^School of Psychology, University College Dublin, Dublin, Ireland

**Keywords:** binge eating disorder, executive function, cognitive flexibility, decision-making, working memory, inhibitory control, problem-solving, set-shifting

## Abstract

**Background:**

Cognitive deficits are implicated in theoretical explanatory models for binge eating disorder (BED). Furthermore, evidence suggest that alterations in executive function may underlie symptoms in BED. The current systematic review and meta-analysis provides an update on executive functioning in individuals with BED.

**Methods:**

Literature searches (up to November 2019) were conducted in electronic databases combining binge eating or BED with executive functions. The Preferred Reporting Items for Systematic Reviews and Meta-Analyses statement guidelines was used. Studies of any design comparing adults with BED with those without BED in executive function domains were selected. Methodological quality of studies was based on the Newcastle-Ottawa scale.

**Results:**

Of 1,983 citations identified, 28 case-control studies met inclusion criteria for this review. Six meta-analyses that examined four domains (decision-making, cognitive flexibility, inhibitory control, and working memory) were conducted. The only meta-analysis to show a significant difference in executive functioning between BED and obese controls was working memory (SMD = 0.32, 95% IC: −0.60, −0.03; *p* = 0.028), with an effect size of small magnitude. Qualitative inspection of the literature indicated mixed findings for control inhibition, decision making and cognitive flexibility in individuals with BED compared to controls (obese or normal weight). In addition, people with BED showed poorer problem solving performance, but similar planning abilities to obese controls.

**Conclusions:**

Individuals with BED were found to show worse performance on working memory tasks compared to obese individuals without the disorder. The findings did not provide definitive evidence of alterations in other aspects of executive functioning. Interest in executive functioning in people with BED is increasing but is limited by insufficient data from small studies with varied methodology. Future studies should focus on using similar tests and outcome measures, in order to enable more pertinent comparisons across studies.

## Introduction

Eating disorders (EDs) affect up to 10% of young women and are associated with significant reductions in quality of life (1, 2). Binge ED (BED) is the most prevalent ED, affecting approximately 2.8% of females and 1% of males (3). BED is characterized by recurrent episodes of binge eating that are not combined with compensatory methods to avoid weight gain. Thus, the majority of BED cases are overweight or obese (4).

The aetiology of BED is not fully understood, nevertheless evidence suggests that inefficiencies in executive functions may underlie symptoms (5). The concept of executive functioning does not have a single definition and is still evolving. However, according to Friedman and Miyake (6), executive functions represent a set of control processes that regulate thoughts and behavior, dysfunctions in which are symptomatic of neuropsychiatric and behavioral disorders. Although there is some debate over which variables should be used to assess executive functioning, inhibitory control, working memory, decision-making, cognitive flexibility, planning, problem-solving are generally well established in neurocognitive research (6–10).

Difficulties to overcome habitual responses rely on top-down processes that may work as risk or maintenance factors for EDs (11). For example, inefficiencies in inhibitory control may be associated with overconsumption of highly palatable foods in individuals with BED (12, 13). Those with BED also display difficulties in decision-making, resulting in a tendency to disregard the negative consequences of binge eating in the long term (14). These deficits could increase the likelihood of binge eating episodes (short term reward)—especially when paired with a lack of adaptive emotion-regulation skills—and lead to weight gain and feelings of guilt (long term consequences) (14, 15). In addition, difficulties in problem solving may make it difficult for individuals with BED to manage and plan ahead for situations in which they are exposed to food-related stimuli (12). In addition, poor working memory, a function that modulates other cognitive abilities such as behavioral inhibition and decision making, may lead to impulsive behaviors such as overeating (16, 17). Lastly, poor cognitive flexibility is associated with difficulties in establishing new patterns of behavior, affecting engagement in therapeutic interventions that focus on changes to well established patterns (12, 18).

Attempts to understand executive functioning in those with EDs are neither exhaustive nor conclusive. One review identified that people with BED had problems in cognitive flexibility compared to obese controls without the disorder (19). Two reviews found poor decision-making performance across individuals with anorexia nervosa, bulimia nervosa and BED compared to healthy controls (20, 21). Conversely, four reviews (11, 22–24) did not find consistent evidence of diminished executive abilities in people with BED. The authors point out that the diversity in methodology, different cognitive tasks and paradigms used, and small sample sizes limit consistent findings.

A systematic review of reviews on neurocognitive functioning in EDs reported that although evidence generally suggests varying patterns of neurocognitive difficulties across EDs, there remain critical limitations regarding the methodological quality of these studies (25). For example, a few of the reviews on the topic did not follow the methodological standards of a systematic review (26, 27), one did not aggregate results in a meta-analysis (11), and others were limited by a specific focus on one task (19–24).

To date, no review and meta-analysis has summarized findings from studies that have examined different domains of executive functioning in individuals with BED.

Therefore, the aim of this systematic review and meta-analysis is to examine whether people with BED perform different to those without the disorder in executive function tasks, and discuss the potential impact of impairments found on binge eating behavior.

## Method

The study was mainly conducted according to the Preferred Reporting Items for Systematic Reviews and Meta-Analyses (PRISMA) protocol (28), although the protocol drafted for this systematic review was developed for the MSc thesis of the first author (MEGC) and not published online before.

### Search Strategy and Study Selection

#### Literature Search

Electronic searches were conducted in Pubmed, PsycINFO, Scopus, and Web of Science databases. Studies published before November 29, 2019 were included. Two terms (and their variations) were combined for the searches: one term was related to the ED diagnosis/behavior (e.g. *BED, binge eating*) and the other referred to executive functions (*executive function, executive control, cognitive control, set-shifting, cognitive flexibility, decision-making, working memory, inhibitory control, problem-solving, attention, and planning*). The descriptors were combined in Boolean operators. In addition, we carried out manual searches of reference lists in order to identify potential additional studies. We did not conduct searches in the gray literature.

#### Study Selection

The eligibility criteria for studies of this review were:

Population: Studies including adults (≥18 years old) diagnosed with BED based on a diagnostic criteria (e.g., DSMIII-R, DSM-IV, or DSM-5), and established through psychiatric clinic interview or any psychiatric diagnostic tool (e.g., Structured Clinical Interview for DSM-Axis I Disorders; Eating Disorders Examination Interview);
Interventions: This review did not explore effects of treatments/interventions, however intervention studies were eligible if they provided baseline comparisons of groups as described below under “comparators” and “outcomes”;
Comparators: Studies that compared participants with BED with participants without BED (either of normal weight or overweight/obese);
Outcomes: Studies that examined performance on an executive function task: set-shifting, cognitive flexibility, decision-making, working memory, inhibitory control, problem-solving and planning;
Studies: Studies with cross-sectional, case-controlled, or clinical trial designs. Additionally, publications in Portuguese, Spanish or English were eligible.

The titles and abstracts that emerged from searches were examined by three independent researchers (MEGC, JK-G, and BSS). Each study that was identified as potentially relevant by at least one of the researchers was read in its entirety to establish whether it met the inclusion criteria of the review. Where any disagreements occurred, a consensus meeting with the three researchers was held to decide on study inclusion.

### Data Extraction

For each study, the first author extracted the following data (presented in **Table 1**): demographic characteristics, body mass index (BMI), psychiatric comorbidities of participants, executive function outcomes examined and their results (mean, standard deviation). In studies with missing information, study authors were contacted by email. The extracted data was checked by a second author before the statistician conducted the meta-analyses.

**Table 1:** Characteristics of included studies—case control design comparing people with binge eating disorder with obese and normal weight controls (n = 28).

Publication	Sample	Female (percentage)	Age (mean ± SD)	BMI (mean ± SD)	Tasks/Outcome variable	Summary of Findings	Quality Score (X/6)
***Decision making***							
Davis et al. (29)	(n = 209) BED: 65 OC: 73 NW: 71	100%	BED: 34.3 ± 6.5 OC: 35.2 ± 6.7 NW: 31.8 ± 6.3	BED: 35.7 ± 9.0 OC: 38.6 ± 7.1 NW: 21.7 ± 1.9	Iowa Gambling Test/ Net score	BED = OC BED < NW	4
Danner et al. (14)	(n = 75) BED: 20 OC: 21 NW: 34	100%	BED: 38.05 ± 10.97 OC: 44.56 ± 13.36 NW: 36.13 ± 14.09	BED: 38.74 ± 6.25 OC: 30.84 ± 3.00 NW: 22.32 ± 1.96	Iowa Gambling Test/ Net score	BED = OC BED < NW	4
Aloi et al. (30)	(n = 90) BED: 45 NW: 45	100%	BED: 30.6 ± 10.9 NW: 25.6 ± 3.5	BED: 35.2 ± 6.5 NW: 20.2 ± 1.6	Iowa Gambling Test/ Net score	BED < NW	4
Blume et al. (31)	(n = 42) BED: 19 OC: 23	BED: 73% OC: 73%	BED: 38.84 ± 9.43 OC: 40.48 ± 10.85	BED: 41.92 ± 5.25 OC: 42.84 ± 4.76	Iowa Gambling Test/ Net score	BED = OC	5
Aloi et al. (32)	(n = 93) BED: 35 OC: 32 NW: 26	BED: 77.1% OC: 50% NW: 69.20%	BED: 44.2 ± 10.7 OC: 49.6 ± 9.9 NW: 46.7 ± 11.1	BED: 38.9 ± 6.9 OC: 36.4 ± 6.8 NW: 23 ± 0.8	Iowa Gambling Test/ Net score	BED < NW BED = OC	5
Dingemans et al. (33)	(n = 81) BED: 25 (no-to-mild depressive symptoms) NW: 56	BED: 88% NW: 87.5%	BED: 32.8 ± 8.5 NW: 36.7 ± 12.3	BED: 38.5 ± 7.4 NW: 23.5 ± 2.8	Iowa Gambling Test/ Net score	BED = NW	6
Svaldi et al. (15)	(n = 35) BED: 17 OC: 18	100%	BED: 42.4 ± 12.3 OC: 38.3 ± 13.1	BED: 32.8 ± 3.54 OC: 30.7 ± 3.92	Game of Dice Task/ Net score	BED < OC	5
Wu et al. (34)	(n = 97) BED: 54 OC: 43	BED: 90.7% OC: 97.7%	BED: 40.07 ± 11.56 OC: 39.81 ± 11.26	BED: 33.95 ± 5.02 OC: 35.08 ± 5.09	Game of Dice Task/ Net score	BED = OC	4
Preuss et al. (35)	(n = 101) BED: 24 OC: 47 NW: 30	BED: 87.5% OC: 97.9% HC: 66.7%	BED: 37.44 ± 12.14 OC: 38.05 ± 9.95 HC: 36.30 ± 12.13	BED: 32.19 ± 4.45 OC: 33.48 ± 3.63 HC: 23.96 ± 2.46	Door Opening Task/ Number of doors	BED = OC = NW	5
Kollei et al. (36)	(n = 144) BED: 48 OC: 48 NW: 48	BED: 77.10% OC: 70.8% NW: 64.60%	BED: 40.69 ± 12.9 OC: 37.94 ± 12.66 NW: 37.67 ± 15.68	BED: 43.31 ± 6.31 OC: 43.58 ± 7.15 NW: 22.07 ± 1.88	Cambridge Gambling Task/ Quality of decision making	BED = OC < NW	5
Grant and Chamberlain (37)	(n = 34) BED: 17 OC: 17	BED: 64.7% OC: 64.7%	BED:25.47 ± 4.82 OC: 23.76 ± 4.09	BED: 33.87 ± 5.08 OC: 31.39 ± 6.28	Cambridge Gamble Task/ Quality of decision making	BED = OC	5
***Inhibitory Control***							
Duchesne et al. (12)	(n = 76) BED: 38 OC: 38	BED: 38.2% OC: 44.7%	BED: 33.29 ± 5.01 OC: 35.42 ± 7.88	BED: 35.89 ± 2.91 OC: 36.60 ± 3.75	The Stroop Test/ Color-word trial: completion time	BED = OC	4
Manasse et al. (5)	(n = 73) BED: 31 OC: 42	100%	BED: 45.06 ± 14.86 OC: 51.09 ± 8.26	BED: 36.84 ± 7.97 OC: 37.85 ± 6.27	Color-Word Interference Task/ Inhibition condition time	BED < OC	4
Eneva et al. (38)	(n = 132) BED-OB: 32 BED-NW: 23 OC: 48 NW: 29	100%	BED-OB: 36.34 ± 2.03 BED-NW: 23.34 ± 0.67 OC: 38.04 ± 1.78 NW: 24.52 ± 1.23	BED-OB: 34.2 ± 0.83 BED-NW: 22.93 ± 0.4 OC: 31.3 ± 0.56 NW: 21.56 ± 0.29	Color Word Interference Task/ Time to completion	BED-OB = BED-NW BED-OB = OC BED-OB = NW	5
Preuss et al. (35)	(n = 101) BED: 24 OC: 47 NW: 30	BED: 87.5% OC: 97.9% HC: 66.7%	BED: 37.44 ± 12.14 OC: 38.05 ± 9.95 HC: 36.30 ± 12.13	BED: 32.19 ± 4.45 OC: 33.48 ± 3.63 HC: 23.96 ± 2.46	Stroop Test/ Reaction time Stop-Signal Task/ Reaction time	BED < NW BED = OC BED > NW BED > OC	5
Dingemans et al. (33)	(n = 81) BED: 25 (no-to-mild depressive symptoms) NW: 56	BED: 88% NW: 87.5%	BED: 32.8 ± 8.5 NW: 36.7 ± 12.3	BED: 38.5 ± 7.4 NW:23.5 ± 2.8	Stroop Test/ Stroop effect	BED = NW	6
Balodis et al. (39)*	(n = 36) BED: 12 OC: 13 NW: 11	BED: 75% OC:38.5% NW: 45.45%	BED: 47.6 ± 12.7 OC: 35.4 ± 9.3 NW: 32.7 ± 11.3	BED: 37.1 ± 3.9 OC: 34.6 ± 4.1 NW: 23.2 ± 1.1	Event-related fMRI Stroop color-word interference task/ Reaction time	BED = OC/NW	5
Lee et al. (40)	(n = 39) BED: 12 NW: 14	100%	BED: 23.6 ± 2.6 NW: 23.3 ± 2.2	BED: 25.6 ± 3.8 NW: 20.4 ± 2.6	Stroop match-to-sample task/ Reaction time	BED = NW	6
Galioto et al. (41)*	(n = 131) BED: 41 OC: 90	BED: 96.3% OC: 83.1%	BED: 43.58 ± 11.45 OC: 41.18 ± 10.40	BED: 45.40 ± 6.12 OC: 44.87 ± 6.58	Verbal interference color/ Words correctly identified	BED = OC	5
Wu et al. (34)	(n = 97) BED: 54 OC: 43	BED: 90.7% OC: 97.7%	BED: 40.07 ± 11.56 OC: 39.81 ± 11.26	BED: 33.95 ± 5.02 OC: 35.08 ± 5.09	Stop-Signal Task/ Reaction time	BED = OC	4
Svaldi et al. (42)*	(n = 60) BED: 31 OC: 29	100%	BED: 45.48 ± 12.77 OC: 40.10 ± 12.11	BED: 35 ± 5.12 OC: 32.99 ± 5.96	Stop-Signal Task/ Reaction time	BED < OC	5
Mole et al. (43)	(n = 60) BED: 30 OC: 30 NW: 30	BED: 56.7% OC: 56.7% NW: 56.6%	BED: 42.92 ± 8.59 OC: 44.06 ± 9.70 NW: 44.12 ± 10.18	BED: 34.68 ± 5.49 OC: 32.72 ± 3.41 NW: 23.86 ± 2.74	Stop-Signal Task/ Reaction time	BED > OC BED = NW	4
Grant and Chamberlain (37)	(n = 34) BED: 17 OC: 17	BED: 64.7% OC: 64.7%	BED:25.47 ± 4.82 OC: 23.76 ± 4.09	BED: 33.87 ± 5.08 OC: 31.39 ± 6.28	Stop-Signal Task/ Reaction time	BED < OC	5
Bartholdy et al. (44)	(n = 39) BED: 11 NW: 28	100%	BED: 28.73 ± 11.33 NW: 24.64 ± 5.14	BED: 28.86 ± 6.92 NW: 22.04 ± 2.03	Stop-Signal Task/ Reaction time	BED = NW	5
Aloi et al. (30)	(n = 90) BED: 45 NW: 45	100%	BED: 30.6 ± 10.9 NW: 25.6 ± 3.5	BED: 35.2 ± 6.5 NW: 20.2 ± 1.6	Hayling Sentence Completion Test/ Part B: time	BED < NW	
Mobbs et al. (18)*	(n = 48) BED: 16 OC: 16 NW: 16	BED: 68.8% OC: 75% NW: 68.75%	BED: 45.1 ± 12.1 OC: 39.3 ± 12.2 NW: 40.2 ± 11.3	BED: 34.6 ± 3.5 OC: 33.6 ± 6.4 NW: 21.3 ± 1.8	Modified affective shifting task/ Errors Commission	BED < OC/NW BED < OC/NW	5
Svaldi et al. (45)*	(n = 92) BED: 29 OC: 33 NW: 30	BED: 100% OC: 100% NW: 100%	BED: 46.83 ± 13.63 OC: 41.97 ± 14.34 NW: 22.00 ± 1.79	BED: 34.73 ± 4.10 OC: 32.98 ± 1.79 NW: 22.00 ± 1.79	Pictorial priming paradigm (in the context of food)/early response inhibition	BED = OC < NW	5
Hege et al. (46)*	(n = 34) BED: 17 OC: 17	100%	BED: 41.88 ± 8.46 OC: 41.35 ± 12.33	BED: 34.01 ± 5.58 OC: 36.52 ± 4.89	Food-related visual Go/No Go task/ Go trial: reaction time No Go trial: reaction time	BED < OC BED < OC	5
Loeber et al. (47)*	(n = 57) BED: 17 OC: 20 NW: 20	BED: 100% OC: 100% NW: 100%	BED: 26.5 ± 3.5 OC: 25 ± 5.2 NW: 23.6 ± 2.0	BED: 39.3 ± 6.0 OC: 33.2 ± 3.2 NW: 22.4 ± 2.1	Go/No Go shifting task/ Commission error	BED > NW food BED < NW neutral BED = OC neutral and food	5
Blume et al. (31)	(n = 42) BED: 19 OC: 23	BED: 73% OC: 73%	BED: 38.84 ± 9.43 OC: 40.48 ± 10.85	BED: 41.92 ± 5.25 OC: 42.84 ± 4.76	Go/No Go shifting task/ Commission error	BED = OC	5
Kollei et al. (36)	(n = 144) BED: 48 OC: 48 NW: 48	BED: 77.10% OC: 70.8% NW: 64.60%	BED: 40.69 ± 12.9 OC: 37.94 ± 12.66 NW: 37.67 ± 15.68	BED: 43.31 ± 6.31 OC: 43.58 ± 7.15 NW: 22.07 ± 1.88	Go/No Go shifting task/ Commission errors in Response to high and low caloric stimuli	BED = OC BED = NW	5
***Working memory***							
Duchesne et al. (12)	(n = 76) BED: 38 OC: 38	BED: 38.2% OC: 44.7%	BED: 33.29 ± 5.01 OC: 35.42 ± 7.88	BED: 35.89 ± 2.91 OC: 36.60 ± 3.75	Digit Span/ Backward: correct answer	BED < OC	4
Reiter et al. (48)	(n = 44) BED: 22 OC: 22	BED: 72.7% OC: 68.2%	BED: 29.0 ± 9.40 OC: 27.8 ± 4.54	BED: 28.27 ± 6.58 OC: 26.06 ± 4.35	Digit Span/ Backward: correct answer	BED = OC	4
Galioto et al. (41)*	(n = 131) BED: 41 OC: 90	BED: 96.3% OC: 83.1%	BED: 43.58 ± 11.45 OC: 41.18 ± 10.40	BED: 45.40 ± 6.12 OC: 44.87 ± 6.58	Digit Span/ Backward: correct answer	BED = OC	5
Dingemans et al. (33)	(n = 81) BED: 25 (no-to-mild depressive symptoms) NW: 56	BED: 88% NW: 87.5%	BED: 32.8 ± 8.5 NW: 36.7 ± 12.3	BED: 38.5 ± 7.4 NW:23.5 ± 2.8	Digit Span/ Backward: correct answer	BED = NW	6
Svaldi et al. (49)	(n = 67) BED: 31 OC: 36	100%	BED: 46.31 ± 14.20 OC: 40.74 ± 13.11	BED: 35.13 ± 5.08 OC: 33.31 ± 6.16	N-Back Task with lures/ Response time	BED < OC	5
Manasse et al. (5)	(n = 73) BED: 31 OC: 42	100%	BED: 45.06 ± 14.86 OC: 51.09 ± 8.26	BED: 36.84 ± 7.97 OC: 37.85 ± 6.27	Pen Letter N-Back Task/ Efficiency score (reaction time and accuracy)	BED = OC	4
Eneva et al. (38)	(n = 132) BED-OB: 32 BED-NW: 23 OC: 48 NW: 29	100%	BED-OB: 36.34 ± 2.03 BED-NW: 23.34 ± 0.67 OC: 38.04 ± 1.78 NW: 24.52 ± 1.23	BED-OB: 34.2 ± 0.83 BED-NW: 22.93 ± 0.4 OC: 31.3 ± 0.56 NW: 21.56 ± 0.29	NIH Toolbox List Sorting Working Memory/Number of items recalled and sequenced correctly	BED-NW < NW BED-OB < NW OC < NW	5
Duchesne et al. (12)	(n = 76) BED: 38 OC: 38	BED: 38.2% OC: 44.7%	BED: 33.29 ± 5.01 OC: 35.42 ± 7.88	BED: 35.89 ± 2.91 OC: 36.60 ± 3.75	Trail Making Test (B)/ Completion time The Rule Shift Cards Test/ Completion time Wisconsin Card Sorting Test/ Perseverative errors	BED = OC BED = OC BED = OC BED < OC	4
Aloi et al. (32)	(n = 93) BED: 35 OC: 32 NW: 26	BED: 77.1% OC: 50% NW: 69.20%	BED: 44.2 ± 10.7 OC: 49.6 ± 9.9 NW: 46.7 ± 11.1	BED: 38.9 ± 6.9 OC: 36.4 ± 6.8 NW: 23 ± 0.8	Trail Making Test (B)/ Completion time	BED < NW BED = OC	5
Reiter et al. (48)	(n = 44) BED: 22 OC: 22	BED: 72.7% OC: 68.2%	BED: 29.0 ± 9.40 OC: 27.8 ± 4.54	BED: 28.27 ± 6.58 OC: 26.06 ± 4.35	Trail Making Test B/ Completion time	BED = OC	4
Eneva et al. (38)	(n = 132) BED-OB: 32 BED-NW: 23 OC: 48 NW: 29	100%	BED-OB: 36.34 ± 2.03 BED-NW: 23.34 ± 0.67 OC: 38.04 ± 1.78 NW: 24.52 ± 1.23	BED-OB: 34.2 ± 0.83 BED-NW: 22.93 ± 0.4 OC: 31.3 ± 0.56 NW: 21.56 ± 0.29	Trail Making Test B/ Completion time	BED-OB < BED-NW/BED-OB < NW/BED-OB = OC	5
Aloi et al. (30)	(n = 90) BED: 45 NW: 45	100%	BED: 30.6 ± 10.9 NW: 25.6 ± 3.5	BED: 35.2 ± 6.5 NW: 20.2 ± 1.6	Wisconsin Card Sorting Test/ Perseverative errors Trail Making Test (B)/ Completion time	BED = NW BED < NW	4
Dingemans et al. (33)	(n = 81) BED: 25 (no-to-mild depressive symptoms) NW: 56	BED: 88% NW: 87.5%	BED: 32.8 ± 8.5 NW: 36.7 ± 12.3	BED: 38.5 ± 7.4 NW:23.5 ± 2.8	Wisconsin Card Sorting Test/ Perseverative errors Trail Making Test/ Part B minus Part A	BED = NW BED = NW	6
Svaldi et al. (15)	(n = 35) BED: 17 OC: 18	100%	BED: 42.4 ± 12.3 OC: 38.3 ± 13.1	BED: 32.8 ± 3.54 OC: 30.7 ± 3.92	Trail Making Test (B)/ Completion time	BED < OC	5
Blume et al. (31)	(n = 42) BED: 19 OC: 23	BED: 73% OC: 73%	BED: 38.84 ± 9.43 OC: 40.48 ± 10.85	BED: 41.92 ± 5.25 OC: 42.84 ± 4.76	Wisconsin Card Sorting Test/ Perseverative errors	BED = OC	5
Kollei et al. (36)	(n = 144) BED: 48 OC: 48 NW: 48	BED: 77.10% OC: 70.8% NW: 64.60%	BED: 40.69 ± 12.9 OC: 37.94 ± 12.66 NW: 37.67 ± 15.68	BED: 43.31 ± 6.31 OC: 43.58 ± 7.15 NW: 22.07 ± 1.88	Intra/Extra-dimensional Set-shift Task/ Shift errors	BED = OC BED = NW	5
Banca et al. (50)*	(n = 63) BED: 32 OC: 31	BED: 56.25% OC: 38.71%	BED: 42.81 ± 8.63 OC: 43.89 ± 9.63	BED: 34.72 ± 5.63 OC: 32.71 ± 3.59	Intra/Extra-dimensional Set Shifting Task/ Number of errors	BED < OC	4
Grant and Chamberlain (37)	(n = 34) BED: 17 OC: 17	BED: 64.7% OC: 64.7%	BED:25.47 ± 4.82 OC: 23.76 ± 4.09	BED: 33.87 ± 5.08 OC: 31.39 ± 6.28	Intra/Extra-dimensional Set-shift Task/ Total errors	BED = OC	5
Manasse et al. (5)	(n = 73) BED: 31 OC: 42	100%	BED: 45.06 ± 14.86 OC: 51.09 ± 8.26	BED: 36.84 ± 7.97 OC: 37.85 ± 6.27	Pen Conditional Exclusion Task/ Perseverative errors	BED = OC	4
Mobbs et al. (18)*	(n = 48) BED: 16 OC: 16 NW: 16	BED: 68.8% OC: 75% NW: 68.75%	BED: 45.1 ± 12.1 OC: 39.3 ± 12.2 NW: 40.2 ± 11.3	BED: 34.6 ± 3.5 OC: 33.6 ± 6.4 NW: 21.3 ± 1.8	Modified affective shifting Task/ Mental flexibility	BED = OC = NW	5
Galioto et al. (41)*	(n = 131) BED: 41 OC: 90	BED: 96.3% OC: 83.1%	BED: 43.58 ± 11.45 OC: 41.18 ± 10.40	BED: 45.40 ± 6.12 OC: 44.87 ± 6.58	Switching of attention/ Completion time	BED = OC	5
Manasse et al. (5)	(n = 73) BED: 31 OC: 42	100%	BED: 45.06 ± 14.86 OC: 51.09 ± 8.26	BED: 36.84 ± 7.97 OC: 37.85 ± 6.27	Tower Task/ Number of move to complete each trial	BED < OC	4
Svaldi et al. (51)*	(n = 55) BED: 25 OC: 30	BED: 100% OC:100%	BED: NA OC: NA	BED: 29.5 ± 3.89 OC: 38.0 ± 8.17	Means-Ends Problem-Solving Procedure (MEPS)/problem solutions	BED < OC	4
Duchesne et al. (12)	(n = 76) BED: 38 OC: 38	BED: 38.2% OC: 44.7%	BED: 33.29 ± 5.01 OC: 35.42 ± 7.88	BED: 35.89 ± 2.91 OC: 36.60 ± 3.75	The Action Program Test/ Number of stages completed	BED < OC	4
***Planning***							
Duchesne et al. (12)	(n = 76) BED: 38 OC: 38	BED: 38.2% OC: 44.7%	BED: 33.29 ± 5.01 OC: 35.42 ± 7.88	BED: 35.89 ± 2.91 OC: 36.60 ± 3.75	The Zoo Map Test/ Planning time-trial 1 Planning time-trial 2 Number of errors-trial 1 Number of errors-trial 2 Time to complete task-trial 1 Time to complete task-trial 2	BED = OC BED = OC BED < OC BED = OC BED = OC BED = OC	4
Eneva et al. (38)	(n = 132) BED-OB: 32 BED-NW: 23 OC: 48 NW: 29	100%	BED-OB: 36.34 ± 2.03 BED-NW: 23.34 ± 0.67 OC: 38.04 ± 1.78 NW: 24.52 ± 1.23	BED-OB: 34.2 ± 0.83 BED-NW: 22.93 ± 0.4 OC: 31.3 ± 0.56 NW: 21.56 ± 0.29	Tower Test (D-KEFS)/Number of moves to complete trial	BED-OB = BED-NW BED-OB = OC BED-OB = NW	5
Galioto et al. (41)*	(n = 131) BED: 41 OC: 90	BED: 96.3% OC: 83.1%	BED: 43.58 ± 11.45 OC: 41.18 ± 10.40	BED: 45.40 ± 6.12 OC: 44.87 ± 6.58	Maze Task/ Number of errors	BED = OC	5
*Mean of included studies (range)*	*BED: 29.3* *OC: 34.4* *NW: 31.8* *(11 – 90)*	*BED: 87.0%* *OC: 83.0%* *NW: 83.9%* *(38.2% – 100%)*	*BED: 38.0* *OC: 39.7* *NW: 32.8* *(22.0 – 51.1)*	*BED: 35.1* *OC: 35.7* *NW: 22.5* *(20.2 – 45.4)*	–	–	

* Not included in the meta-analyses.

Findings with an > indicate a favorable result for the BED group, while those with an < indicate a favorable result for the comparison group. Results with an = indicate no significant differences between groups.

BMI, body mass index; BED, binge eating disorder; D-KEFS, Delis-Kaplan Executive Function System; OB, obese; OC, obese control; NIH, National Institutes of Health; NA, not available; NW, normal weight control; SD, standard deviation.

### Quality Assessment

A standardized checklist to identify risk of bias was used to assess the quality of included studies. The checklist was based on the Newcastle-Ottawa Scale (52) and adapted by authors of this study. Only the quality items of the first two aspects (‘selection' and ‘comparability') were considered, given that the third domain (“exposure”) was not pertinent to the focus of studies included of this review. Ratings were summed to provide a total score with a maximum value of six: four points for sample selection and assessment of potential for selection biases, and two points for comparability and controlling for confounding factors. Quality levels of evidence were defined as high (5–6 points); medium (3–4 points), and low (1–2 points). Studies were excluded if they scored in the low range. The quality assessment was conducted by the first author and revised by the last author.

### Quantitative Data Synthesis

Meta-analyses were carried out aggregating results from studies that examined the same executive function subdomain. Studies using different neuropsychological tests to examine the same executive function were included in the same meta-analysis. However, separate meta-analyses were run for studies using reaction time as the outcome measure, distinct from those that used a “score” to measure the performance of the same executive function. Additionally, studies would be separated in different meta-analyses where paradigms used to examine a same domain were considered too different.

In studies where three groups were being compared (i.e., BED, normal weight controls, and obese/overweight controls), priority was given for comparisons of the BED results with those of the overweight control group, since the majority of people with BED are obese or overweight and there was interest in examining differences that could be associated with the ED itself, i.e., over and above the potential impact of the weight status (obese).

The Standardized Mean Difference (SMD) was used to calculate the mean differences between groups. The effect sizes were calculated as Hedges' g (a variation of Cohen's d that corrects for biases due to small sample sizes), and 95% Confidence Intervals (CI) were reported. The magnitude of the effects was interpreted as small (0.15–0.45), medium (0.5–0.75), and large (≥0.8).

Heterogeneity among studies was assessed using the Cochran's Q test (53). An additional measure of heterogeneity or inconsistency across studies was also applied, the Higgins and Thompson *I^2^* index [*I^2^* = (Q – df)/Q] (54). As a sample size independent measure of the inconsistency of effect sizes across studies, *I^2^* is more powerful with small sample sizes, compared to Cochran's Q test (54). The *I^2^* index describes the percentage of total variation across studies that is due to heterogeneity rather than sampling error, and ranges from 0% (no inconsistency) up to 100% (high heterogeneity). The studies are considered heterogeneous when the variability between them has a nonrandom origin, with values >50% considered as moderate heterogeneity and >75% considered high (54). The random effects model was always preferred when significant heterogeneity is observed (and *I^2^* > 50%) between the studies, and the fixed effects model when the heterogeneity was considered low and not significant.

The statistical program STATA 12 was used to carry out all analyses. In the forest plots, the case group refers to people with BED and the control group refers to obese controls. Forest plots present the results (SMD and CI) for each study in the meta-analysis and the measure of meta-analytic effect.

Finally, despite relatively few articles included in each meta-analysis, publication bias was assessed through visual inspection of funnel plot asymmetry (55) and by the corresponding statistical analogues: Begg's adjusted rank test (56) and Egger's test (57). The funnel plots of all meta-analyses are in **Supplementary Material**.

## Results

### Included Studies

**Figure 1:**
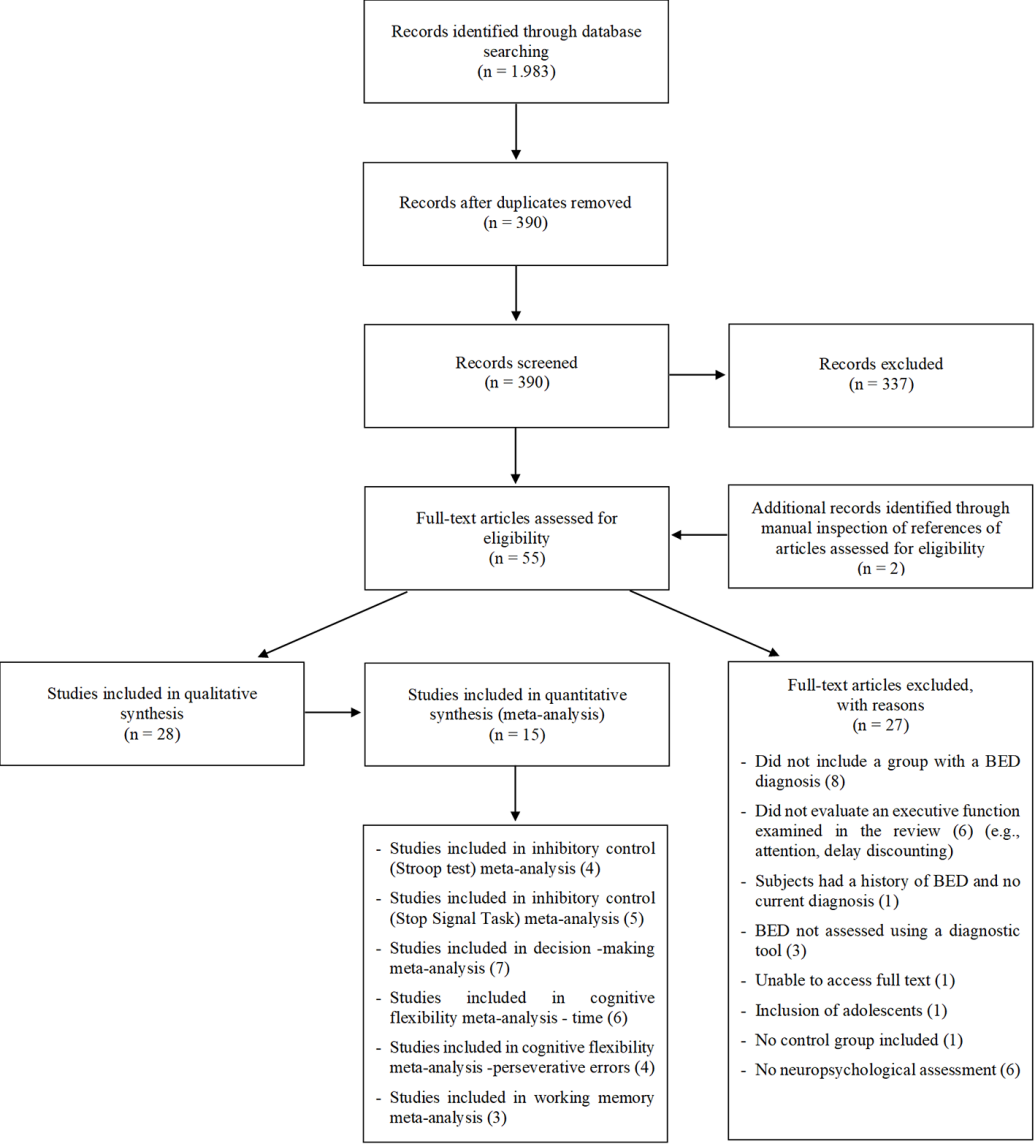
Preferred Reporting Items for Systematic Reviews and Meta-Analyses (PRISMA) flow chart of study Inclusion.

Out of a total of 1.983 records, 390 were selected for abstract reading after duplicates were removed. After screening of abstracts, 53 potentially eligible articles for full-text reading were identified. Two additional papers were identified through manual inspection of reference lists of eligible articles. Evaluation of these full-texts resulted in 28 studies being included in this review (see **Figure 1**). All included studies were cross-sectional. Fifteen (5, 12, 14, 15, 30–32, 34–37, 43, 47–49) of the 28 studies provided sufficient data to be included in at least one of the meta-analyses conducted. Missing data were requested from authors of 15 original articles and 6 replied to requests, providing unpublished data (15, 30, 36, 39, 42, 58). Regarding psychiatric comorbidities, 25 studies reported that the BED group had some comorbidity, 2 did not provide this information (32, 48) and 1 (40) reported exclusion of individuals with a current or past psychiatric disorder. Not all studies specified the type of comorbidities.

In the methodological quality assessment, 18 studies received a “high” quality score, and 10 received scores within the “medium” range. No studies were therefore excluded due to low methodological quality. Study characteristics are summarized in **Table 1**. Some studies examined multiple executive functions and for that reason are listed several times.

### Data Synthesis and Meta-Analysis of the Executive Functions

In this section, the results of meta-analyses are reported first, followed by a qualitative discussion of results from studies that could not be included in meta-analyses.

Separate analyses were conducted for studies using Stroop and Stop-Signal paradigms to measure inhibitory control, because of differences between these neurocognitive tasks. Similarly, in the cognitive flexibility domain, independent analyses were conducted for time taken to perform the Trail Making Test (TMT) and The Rule Shift Cards Test, and the number of perseverative errors in the Wisconsin Card Sorting Test (WCST), Penn Conditional Exclusion Task (PCET) and Intradimensional/Extradimensional Set-Shift task (IED).

#### Studies Not Included in Meta-Analyses

Amongst the studies selected for the systematic review (30), 13 were not included in any of the meta-analyses conducted. Reasons for not combining data from these studies were: the use of instruments that provided scores in a different manner from the others (18, 39, 41, 42, 45–47, 51), lack of raw data (means and standard deviation) in published material which was not obtained from authors upon request (50), and studies that compared BED and normal weight control groups (32, 33, 40, 44). This last group of studies (which compared BED and normal weight control groups) could not be included in meta-analyses as there was no domain for which at least 3 studies examined the same function using comparable measures, which would enable aggregation of results. However, they were included in the qualitative description of findings. Additionally, meta-analyses could not be conducted for studies investigating planning or problem-solving, as they did not use comparable measures. Findings from all studies that examined executive function domains not aggregated in meta-analyses for any of the above mentioned reasons are presented in **Table 1**.

#### Inhibitory Control

##### Stroop Test

Four studies (5, 12, 35, 38) were included in a meta-analysis comparing Stroop test performance in individuals with BED to obese controls (n = 382). No significant differences were found between the BED group and the obese control group, with a pooled effect size of 0.18 (95% IC: −0.05, 0.42, *z* = 0.154, *p* = 0.123). No evidence of significant heterogeneity, *X*^2^ (3, n = 250) = 3.02, *p* = 0.388, *I^2^* = 0.70%) or of publication bias was observed (Begg's test *z* = 0.00, *p* = 1.000 and Egger t (1) = 0.58, *p* = 0.62).

##### Stop Signal Task

Five studies (23, 35, 37, 43, 49) were included in a meta-analysis examining inhibitory control using the Stop Signal Task (n = 359). No significant differences between the BED group and the obese control group were found, with a pooled effect size of 0.17 (95% IC: −0.40, 0.74, *z* = 0.58, *p* = 0.562). The Cochran Q test revealed significant heterogeneity across studies, *X^2^* (4, n = 622) = 24.51, *p <*0.001, *I^2^* = 83.70%). No evidence of publication bias was observed (Begg's test *z* =0.98, *p* = 0.327 and Egger t (1) = 0.81, *p* = 0.478).

Four studies using different Go/No-Go paradigms reported mixed findings (31, 36, 46, 47). Hege et al. (46) concluded individuals with BED showed worse performance than obese controls. The other three studies did not find any evidence of altered performance in individuals with BED compared to obese (31, 36, 47) or normal weight controls (36, 47).

Mobbs et al. (18) found that inhibition problems on a mental flexibility task were more severe in the BED group compared to obese and normal weight controls. Galioto et al. (41) found no differences between individuals with BED and an obese control group in a verbal interference color test. Balodis et al. (39) used an event-related fMRI Stroop color-word interference task, finding no significant group differences to congruent or incongruent stimuli in individuals with BED compared to obese and normal weight controls. Compared with normal weight controls, individuals with BED showed worse performance on the Hayling Sentence Completion Task (HSCT) in one study (32), but no differences in another 3 studies using the Stroop test (33), the Stop-Signal task (44) and Stroop match-to-sample task (40). Svaldi et al. (45) used a Pictorial priming paradigm and found that individuals with BED and obese controls demonstrated poorer performance compared to normal weight controls, but did not differ from one another.

#### Working Memory

**Figure 2:**
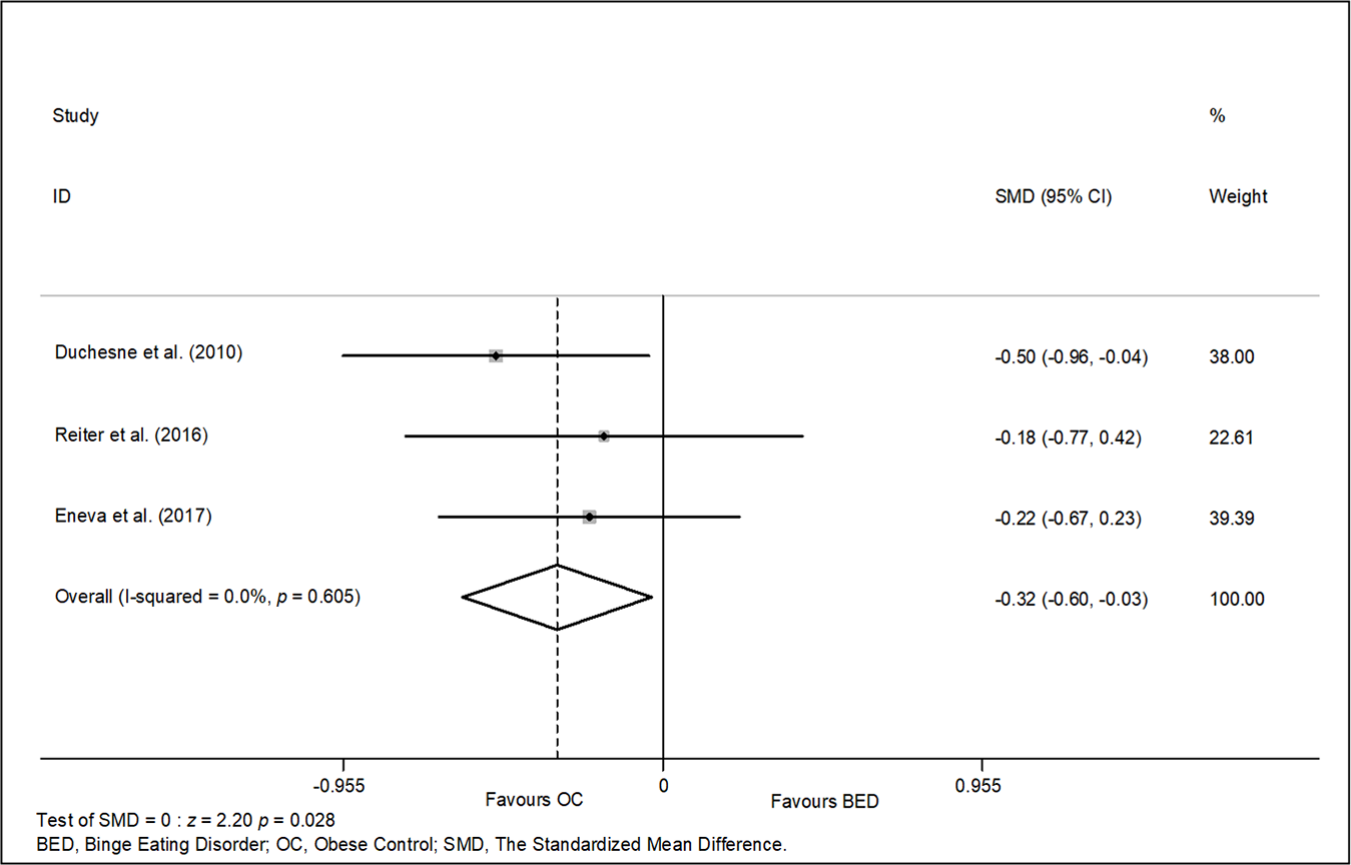
Meta-analysis of studies examining working memory function (three studies, n = 200).

Three studies (n = 200) were included in a meta-analysis examining working memory (12, 38, 48) based on performance on the Digit Span test (backward) and the NIH toolbox test (See **Figure 2**). A significant difference of small magnitude favouring the obese control group was found, with a pooled effect size of −0.32 (95% IC: −0.60, −0.03; *p* = 0.02). There was no evidence of significant heterogeneity, *X^2^* (2, n = 200) = 1.01, *p* = 0.605, *I^2^* = 0.0%), or publication bias (Begg's test *z* = 0.00, *p* = 1.000 and Egger t (1) = 0.54, *p* = 0.686).

Four additional studies that were not included in the meta-analysis reported mixed findings. Three showed no significant differences between individuals with BED and obese (5, 41) or normal weight controls (33), however Svaldi et al. (49) found that BED performed worse than obese controls (see **Table 1**).

#### Decision-Making

Seven studies (n = 649) were included in a meta-analysis examining decision-making based on the net score in the Game of Dice Task (GDT) (15, 23), the Iowa Gambling Test (IGT) (14, 29, 31, 32) and the Door Opening task (35). No significant difference between individuals with BED and obese controls was found (SMD = −0.08; 95% IC: −0.29, 0.13; *p* = 0.467). There was no evidence of heterogeneity between studies, Cochran Q test, *X^2^* (5, n = 649) = 6.07, *p* = 0.300, *I^2^* = 17.60%). There was no evidence of publication bias (Begg's test *z* = −0.94, *p* = 0.348 and Egger t (1) = −1.41, *p* = 0.231).

Studies not included in the meta-analysis (30, 33, 36, 37) reported mixed findings. People with BED showed higher rates of decision-making impairments compared to normal weight controls in three studies. On the other hand, others reported no significant differences in this domain between BED and obese controls (36, 37) and normal weight controls (33).

#### Cognitive Flexibility

##### Time: TMT and the Rule Shift Cards Test

Data from 6 studies (n = 299) were included in a meta-analysis examining cognitive flexibility based on the measure “time” from the TMT part B. No significant difference between groups was found (SMD = 0.19; 95% IC: −0.01, 0.40, *z* = 1.84, *p* = 0.065). The Cochran Q test did not reveal significant heterogeneity, *X^2^* (5, n = 299) = 9.61; *p* = 0.087, *I^2^* = 48%). Additionally, studies included did not show any evidence of publication bias (Begg's test *z* =1.88, *p* = 0.060 and Egger t(1) = 1.84, *p* = 0.139).

##### Perseverative Errors: WCST, PCET, and IED

Four studies (n = 287) were included in a meta-analysis examining cognitive flexibility based on perseverative errors (5, 12, 31, 36). Groups did not significantly differ on this measure (SMD = 0.10; 95% IC: −0.32, 0.51, *p* = 0.642). Significant heterogeneity among studies was observed (*I^2^* = 66.90%, Cochran Q Test, *X^2^* (3, n = 287) = 9.06, *p* = 0.02). Included studies did not show any evidence of publication bias (Begg's test *z* = 0.34, *p* = 0.73 and Egger t(1) = −1.15, *p* = 0.36).

Six additional studies not included in the meta-analysis reported mixed findings. In three studies, there was no evidence of altered performance in the BED group compared to obese (18, 37, 41) or normal weight controls (18, 33). In one study (56), individuals with BED showed poorer performance compared to normal weight controls on all indexes of the WCST, apart from perseverative errors. In another study (50), the BED group showed worse performance than normal weight controls in the Intra/Extra-dimensional set shifting task.

#### Problem-Solving (Only Qualitative Analyses)

Three studies by Duchesne et al. (12), Manasse et al. (5), and Svaldi et al. (51), evaluated problem-solving using the Action Program Test, the Tower Task and Means-Ends Problem-Solving Procedure respectively. Individuals with BED demonstrated poorer performance compared to obese controls.

#### Planning (Only Qualitative Analyses)

Across different tasks (Zoo map, D-KEFS tower, and maze task), three studies found no significant differences in planning ability in individuals with BED compared to obese controls (12, 38, 41).

## Discussion

The present systematic review explored a broad range of executive functions in patients with BED compared to obese and normal-weight controls, but the meta-analyses conducted only compared BED with obese controls. In four of these domains (decision-making, cognitive flexibility, inhibitory control and working memory) it was possible to aggregate data in six meta-analyses. In five out six meta-analyses no evidence of altered executive functioning in individuals with BED was found. Overall, these meta-analyses were limited by small numbers of combined studies (maximum 6 for decision-making). Qualitative inspection of the literature indicated mixed, inconclusive findings for control inhibition, decision making and cognitive flexibility in individuals with BED compared to controls (obese or normal weight), we will discuss it later. Only one small meta-analysis (n = three studies, 200 participants in total) suggested poorer working memory performance in people with BED compared to obese individuals without BED (29, 30, 33), with a small effect size.

As far as we know, no previous meta-analysis has examined working memory in people with BED. However, studies that examined working memory and were not included in the meta-analysis were not supportive of differences between BED and obese or normal weight controls (5, 33, 41). Conversely to our meta-analysis findings, and in line with three previous reviews, there was inconsistent evidence of impairments in working memory in people with BED compared to obese controls (11, 25, 59). Working memory refers to the cognitive process that maintains, manipulates and updates incoming information in real time to guide proximal decision-making and behavioral responses (60, 61). This function seems to play an important role in the successful self-regulation of eating behavior and body weight (62, 63). Some studies have pointed out a possible association between working memory alterations and binge eating behavior (12, 38). That is, poor working memory may impair the capacity to keep track of ongoing impulsive acts (i.e., binging) (12), and may lead to the maintenance of binge-eating by allowing distractors to overwhelm self-regulation goals (38). Despite these theories, it is important to be cautious with any attempts to associate cognition and eating behavior. Working memory is a “fluid” cognitive ability, meaning that it is susceptible to changes due to factors such as sleep alterations, medication use, or nutrition (64). For instance, a previous study in obese individuals reported that obesity was associated with deficits in working memory. Poor working memory was associated with more consumption of fatty foods, potentially contributing to the development and maintenance of obesity (65). Thus, poor performance in working memory tasks could be attributed to several factors beyond BED. Additionally, it is important to mention that the studies included in this and in other reviews used different cognitive tasks (e.g., Digit Span and N-Back Task) to assess working memory (see **Table 2** in **Supplementary Materials**). In their review, Redick and Lindsey (66) pointed out that Span and N-back tasks measure different cognitive processes. Therefore, different tasks can be used to evaluate different working memory components, and combination of results of these tasks may provide biased results and inappropriate interpretation (67). In our meta-analysis of working memory, we were careful not to combine these two tests.

The present review did not provide evidence to suggest inhibitory control is altered in BED. This is in contrast with studies reporting impulsive behavior and difficulties in controlling behavioral responses in comparison to obese individuals without the condition (5, 39). Our findings are in line with a systematic review of reviews (25), which found no differences between individuals with BED and obese or normal weight control groups using food-related stimuli for general inhibitory control. Similarly, our meta-analysis of studies using the Stop-Signal Task did not find a significant difference in performance in individuals with BED compared to obese controls. This finding has been corroborated by other reviews (11, 22, 24). Thus, although a few studies (18, 32, 45, 46) have reported that BED impacts inhibitory control task performance, this has not been supported by four reviews that examined this domain. It is worth mentioning that the Stroop task was originally constructed to assess cognitive interference and not inhibitory control, which limits the interpretation of results from this meta-analysis and might explain our null findings.

Contrary to our expectations, the decision-making and cognitive flexibility meta-analyses did not find significant differences in individuals with BED compared to controls. Nonetheless, in relation to the decision-making domain, the different types of tasks used in studies may have contributed to the overall null findings. The IGT (14, 29, 30) evaluates decision-making under ambiguity, while the GDT assesses decision-making under risk (15, 34), where risk is presented *a priori* and the subject can calculate the chances of winning or losing in each bet (68). It is not clear whether individuals with BED show poorer performance in a more intuitive-experiential mode, which is associated with automatic and emotional processing (as in IGT), than in a more rule-governed mode (as in GDT). In regard to cognitive flexibility, some of the tasks used in studies are thought to be multi-determined tasks, i.e., they reflect a wide variety of cognitive processes, rather than flexibility only (69). However, this review has assumed that the measures included (i.e. time to complete the task, and number of perseverative errors) are reliable measures of flexibility. Under this assumption, the two meta-analyses that combined results of these measures separately did not find significant differences between individuals with BED and obese controls. Thus, our results do not support reduced cognitive flexibility in people with BED, a finding that does not corroborate results of a previous systematic review (19). The differing results are likely due to the very small number of studies included in the previous review (n = 2). However, our null findings are in line with three studies included in this review that were not included in the meta-analysis (18, 33, 41).

Problem-solving has been scarcely examined in individuals with BED, with only three studies identified in this review. Findings from these studies suggest poorer problem-solving ability in people with BED compared to obese controls (5, 12, 51). Similarly, few studies (n = 3) examined planning ability. Findings from these studies do not support altered planning abilities in individuals with BED (12, 38, 41). It is important to note that the results from the meta-analyses suggest that individuals with BED do not have more deficits than obese controls, however this does not necessarily mean performance is normal or similar to a healthy control group.

The qualitative examination of findings from studies comparing people with BED and obese or normal weight controls suggest: (a) mixed findings in decision-making, cognitive flexibility and inhibitory control; (b) no differences between groups in planning. Additionally, individuals with BED showed worse problem solving abilities compared to obese controls. A hypothesis to be tested in the future research is whether p-hacking could explain these confounding findings in isolated studies.

This systematic review highlights the need for more studies examining neuropsychological performance in people with BED. The findings have several important implications. Firstly, considerable methodological heterogeneity was found among studies, also pointed out in a previous review (11). Studies used different tests and outcome measures to evaluate the same function. Secondly, there is no standardization for control groups. Obese individuals can be considered as a better control sample for future studies in the field, as BED is commonly associated with obesity. However, one-third of people with BED are of normal weight (4). Thus, it would be of interest to compare people with BED with controls of both nutritional status, and either control for the impact of BMI in analyses, or compare people with similar BMIs. Other aspects that might have contributed to apparent inconsistencies in study findings but were not systematically reported by studies include: (a) the impact of psychiatric comorbidities of patients with BED, not usually controlled in studies (considering the fact that many different psychiatric disorders may be associated with some level of cognitive dysfunction) (70); (b) treatments that might interfere with neurocognition, such as psychiatric medication use and psychological treatments, and (c) sample type (clinical or community sample). Finally, it is important to clarify that these issues relating to methodological heterogeneity among studies are different from the criteria used for the quality assessment in this review.

### Strengths and Limitations of This Review

Strengths of this systematic review include: a larger sample of overweight/obese individuals (as in Smith et al. (25) review); the extension of previous meta-analyses that focused on one specific executive function (21) or on one selected executive function task (22), as this review covered a broader spectrum of executive functions and took into account a wider range of executive function measures; and the fact that it is possibly the first study to perform meta-analysis of working memory measures in people with BED. Besides, studies were carefully examined by a standardized checklist to identify risk of bias prior to meta-analyses.

A limitation of our review was the small number of articles included, particularly in meta-analyses, restricting the strength of the evidence that emerges from the results. This was due in part to the limited number of studies available in the field to date. As mentioned above, other methodological differences across studies also limited combination and interpretation of findings. That is, even when trying to aggregate results from studies that examined the same domain, the variety of tests used and the cognitive processes that these tests reflect made comparisons difficult. It is also important to note that we were not able to compare BED with normal weight controls in meta-analyses. Comparisons between BED and obese controls are somewhat limited, as obesity itself is also associated with difficulties in several executive functions.

### Clinical Implications

Cognitive deficits are implicated in theoretical explanatory models of BED, such as the transdiagnostic food addiction model (71). To examine whether differences in executive function are of causal significance, further longitudinal studies and investigation as to whether targeting them in treatment provides symptomatic benefit is required. Treatments such as inhibition training show some potential. For example, targeting impulsive actions (such as loss of control overeating) through strengthening inhibitory processes during training is a potentially valuable technique (72). Neuromodulation approaches may also work through these mechanisms. It is possible that a personalized psychiatry approach may be needed in which treatments are tailored to the underlying intermediate phenotype. More treatment studies which examine executive processes as moderators or mediators are therefore required. The development of a standardized cognitive battery through joined forces of experts from both fields (ED and neuropsychology) can potentially allow reproducibility and reduce inconsistencies of findings (73), as has been developed in the schizophrenia field (e.g., MATRICS battery) (74).

## Conclusion

In conclusion, the findings from our meta-analysis suggest that individuals with BED may show alterations in working memory, relative to obese people without the disorder (with an effect size of small magnitude). In other domains, the meta-analyses suggest that patients with BED do not show more difficulties in executive functioning than obese controls. However, this does not necessarily indicate similar performance to healthy, nonobese controls. It is hoped that the findings from this review stimulate further research with stronger designs in the field, as well as the development of therapeutic approaches that take patients' cognitive profile into account.

## Data Availability Statement

All datasets generated for this study are included in the article/**Supplementary Material**.

## Ethics Statement

The study was approved by the ethics committee of Universidade Federal de São Paulo (case 9812050316).

## Author Contributions

MC performed the searches and data extraction and wrote the manuscript. AB, BS and JK-C helped with titles and abstracts and papers selection. AC and FS contributed with concept, protocol writing, revision, and interpretation of findings. JK-G also contributed with revision and edit of the manuscript.

## Conflict of Interest

The authors declare that the research was conducted in the absence of any commercial or financial relationships that could be construed as a potential conflict of interest.

## Supplementary Material

Supplementary Figure 1 The updated search of the systematic review.

Supplementary Figure 2 Forest plot of standardized mean effect size of differences (SMD) in decision-making between individuals with binge eating disorder (BED) and obese healthy controls (OC).

Supplementary Figure 3 Funnel plot of studies included in the decision-making meta-analysis.

Supplementary Figure 4 Forest plot of standardized mean effect size of differences (SMD) in cognitive flexibility between individuals with binge eating disorder (BED) and obese healthy controls (OC). (Wisconsin Card Sorting Test (WCST), Penn Conditional Exclusion Task (PCET) and Intradimensional/ Extra-dimensional Set-Shift task (IED)/perseverative errors).

Supplementary Figure 5 Funnel plot of studies included in the cognitive flexibility meta-analysis (Wisconsin Card Sorting Test (WCST), Penn Conditional Exclusion Task (PCET) and Intradimensional/ Extra-dimensional Set-Shift task (IED)/perseverative errors).

Supplementary Figure 6 Forest plot of standardized mean effect size of differences (SMD) in cognitive flexibility between individuals with binge eating disorder (BED) and obese healthy controls (OC). (Trail Making Test (TMT) and The Rule Shift Cards Test / time).

Supplementary Figure 7 Funnel plot of studies included in the cognitive flexibility meta-analysis (Trail Making Test (TMT) and The Rule Shift Cards Test / time).

Supplementary Figure 8 Funnel plot of studies included in the working memory meta-analysis.

Supplementary Figure 9 Forest plot of standardized mean effect size of differences (SMD) in inhibitory control between individuals with binge eating disorder (BED) and obese healthy controls (OC) (Stroop test).

Supplementary Figure 10 Funnel plot of studies included in the inhibitory control meta-analysis (Stroop Test).

Supplementary Figure 11 Forest plot of standardized mean effect size of differences (SMD) in inhibitory control between individuals with binge eating disorder (BED) and obese healthy controls (OC). (Stop Signal Task).

Supplementary Figure 12 Funnel plot of studies included in the inhibitory control meta-analysis (Stop Signal Task).

Supplementary Figure 13 Data extraction form.

Supplementary Table 1 Characteristics of excluded studies.

Supplementary Table 2 Studies included in each executive function subdomain and in meta-analyses.
