# Supplementary material for: Scrutinizing Domains of Executive Function in Binge Eating Disorder: A Systematic Review and Meta-Analysis
Source: Front Psychiatry. 2020 Apr 17;11:288. doi: 10.3389/fpsyt.2020.00288 (PMC7181673; doi:10.3389/fpsyt.2020.00288)
Supplement: Supplementary Figure 1 — The updated search of the systematic review. [file DataSheet_1.pdf]

![frontiers]

Supplementary Material

Database: Scopus

Date of search: 19.11.19
Data parameters: none
Search Strategy: (((binge eating disorder) OR binge eating)) AND ((((((((((executive function) OR executive control) OR cognitive control) OR set shifting) OR cognitive flexibility) OR decision making) OR working memory) OR inhibitory control) OR problem solving) OR attention) OR planning)

Database: Web of Science

Date of search: 19.11.19
Data parameters: none
Search Strategy: (((binge eating disorder) OR binge eating)) AND ((((((((((executive function) OR executive control) OR cognitive control) OR set shifting) OR cognitive flexibility) OR decision making) OR working memory) OR inhibitory control) OR problem solving) OR attention) OR planning)

Database: PubMed

Date of search: 21.11.19
Data parameters: none
Search Strategy: (((binge eating disorder) OR binge eating)) AND ((((((((((executive control) OR cognitive control) OR executive function) OR set shifting) OR decision making) OR working memory) OR inhibitory control) OR problem solving) OR attention) OR cognitive flexibility) OR planning)

Database: PsycINFO, PsycARTICLES, PsycBOOKS

Date of search: 20/11/2019
Data parameters: From 01/01/2009 to 20/11/2019
Search Strategy: Abstract: binge eating disorder OR Abstract: binge eating AND Abstract: executive control OR Abstract: cognitive control OR Abstract: set shifting ORAbstract: decision making OR Abstract: working memory OR Abstract: inhibitory control OR Abstract: problem solving OR Abstract: attention ORAbstract: cognitive flexibility AND Abstract: Planning AND Year: 2009 To 2019

Supplementary Figure 1. Review search update 2019

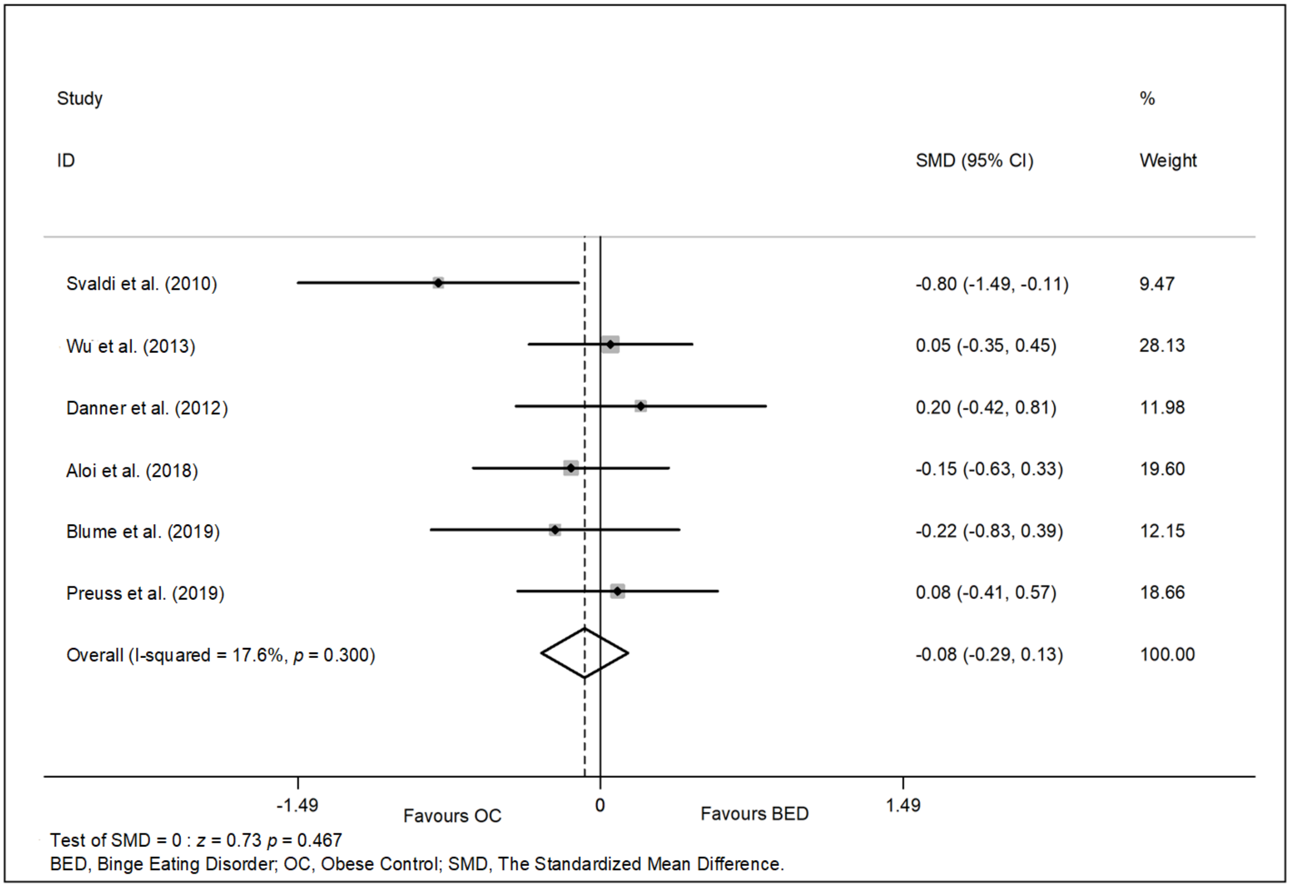

Supplementary Figure 2. Meta-analysis of studies examining decision-making function

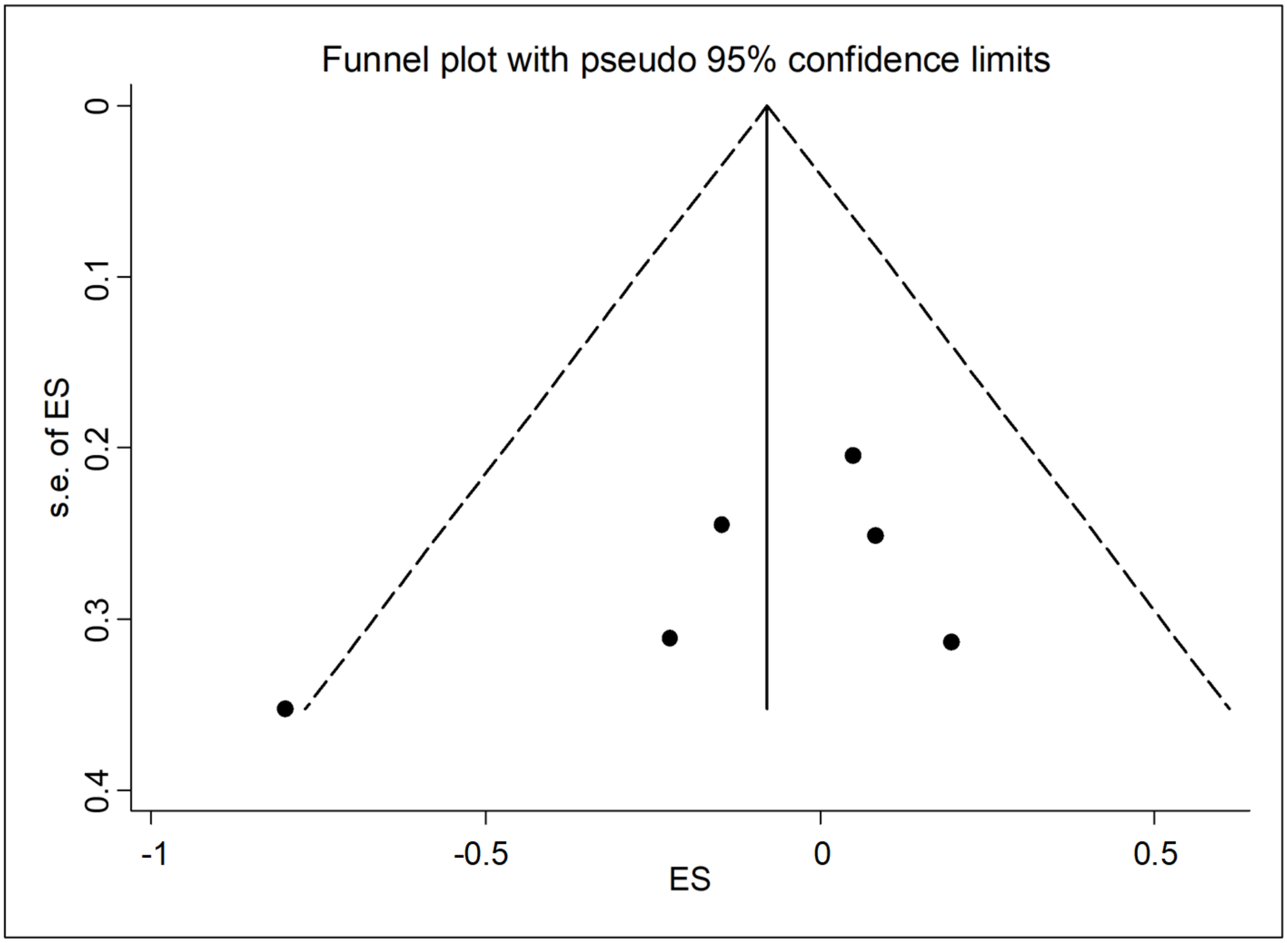

Supplementary Figure 3. Funnel plot of studies included in the decision-making meta-analysis.

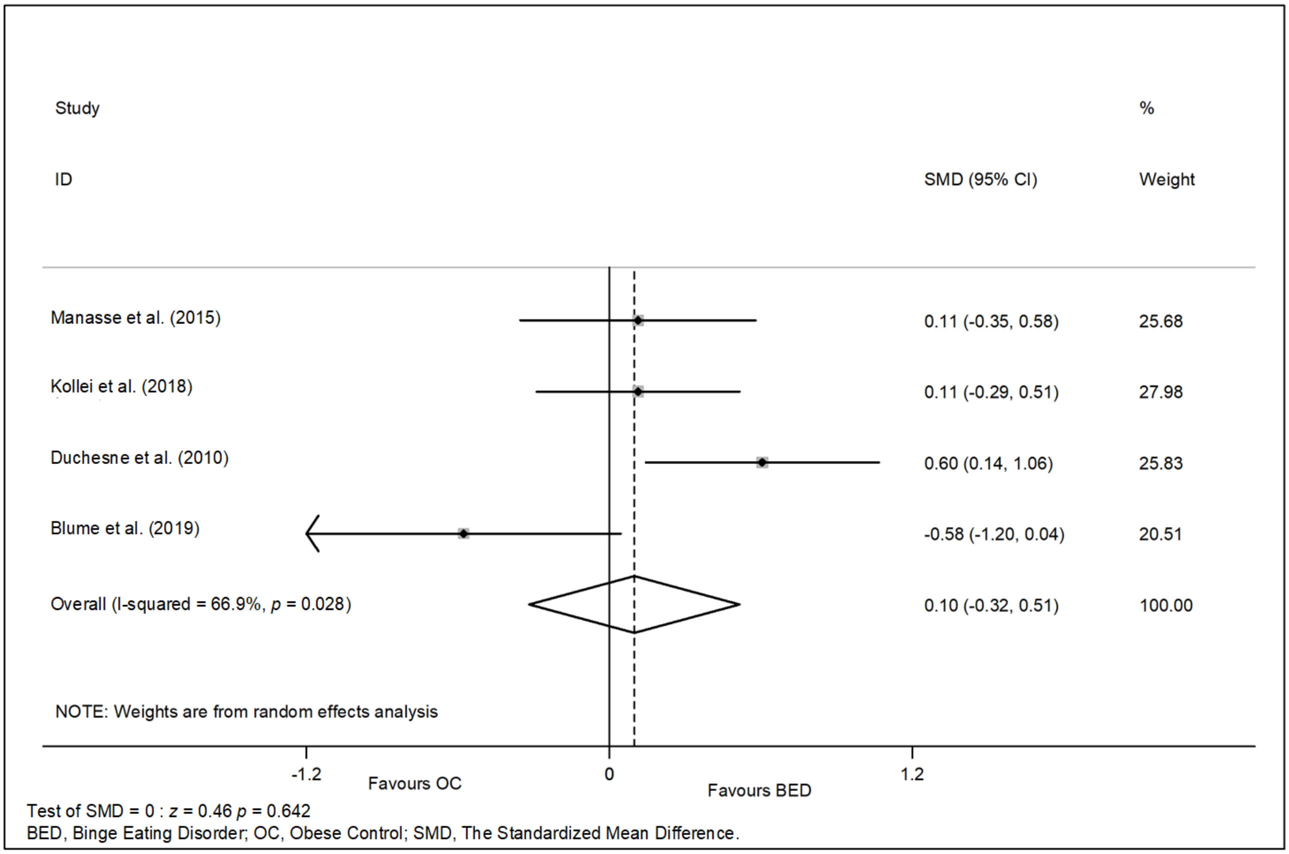

Supplementary Figure 4. Forest plot of standardized mean effect size of differences (SMD) in cognitive flexibility between individuals with binge eating disorder (BED) and obese healthy controls (OC). (Wisconsin Card Sorting Test (WCST), Penn Conditional Exclusion Task (PCET) and Intra-dimensional/Extra-dimensional Set-Shift task (IED)/perseverative errors)

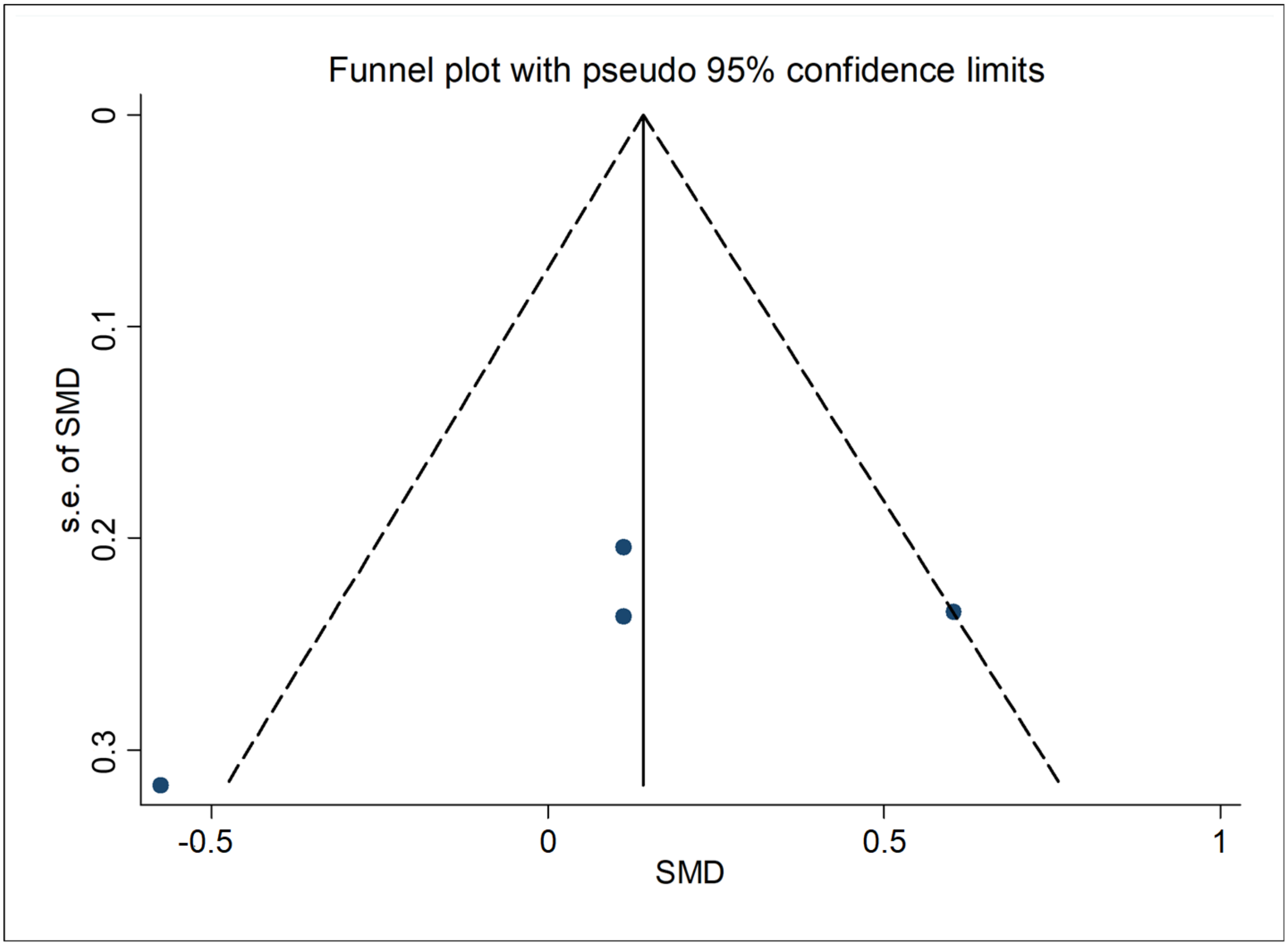

Supplementary Figure 5. Funnel plot of studies included in the cognitive flexibility meta-analysis (Wisconsin Card Sorting Test (WCST), Penn Conditional Exclusion Task (PCET) and Intra-dimensional/Extra-dimensional Set-Shift task (IED)/perseverative errors)

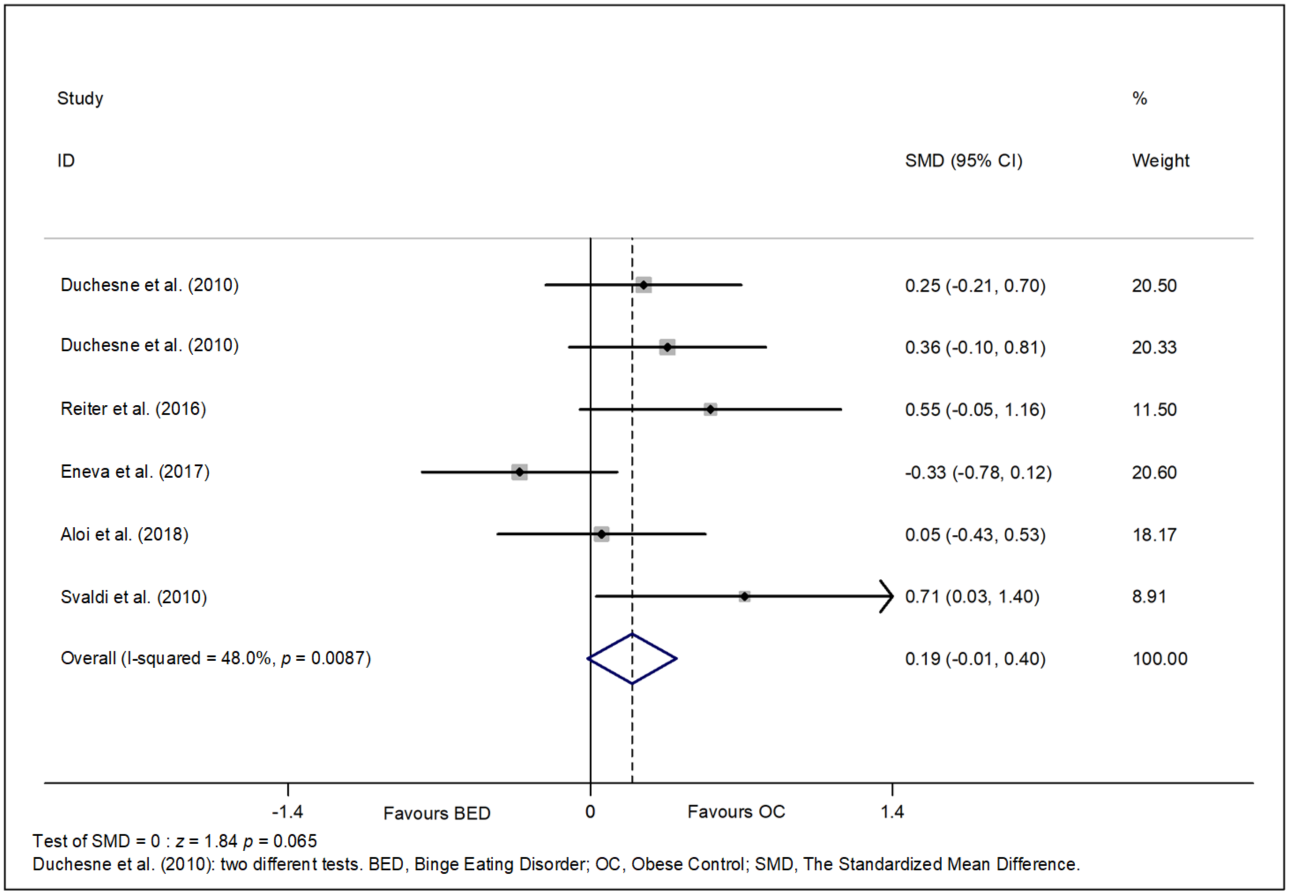

Supplementary Figure 6. Forest plot of standardized mean effect size of differences (SMD) in cognitive flexibility between individuals with binge eating disorder (BED) and obese healthy controls (OC). (Trail Making Test (TMT) and The Rule Shift Cards Test / time)

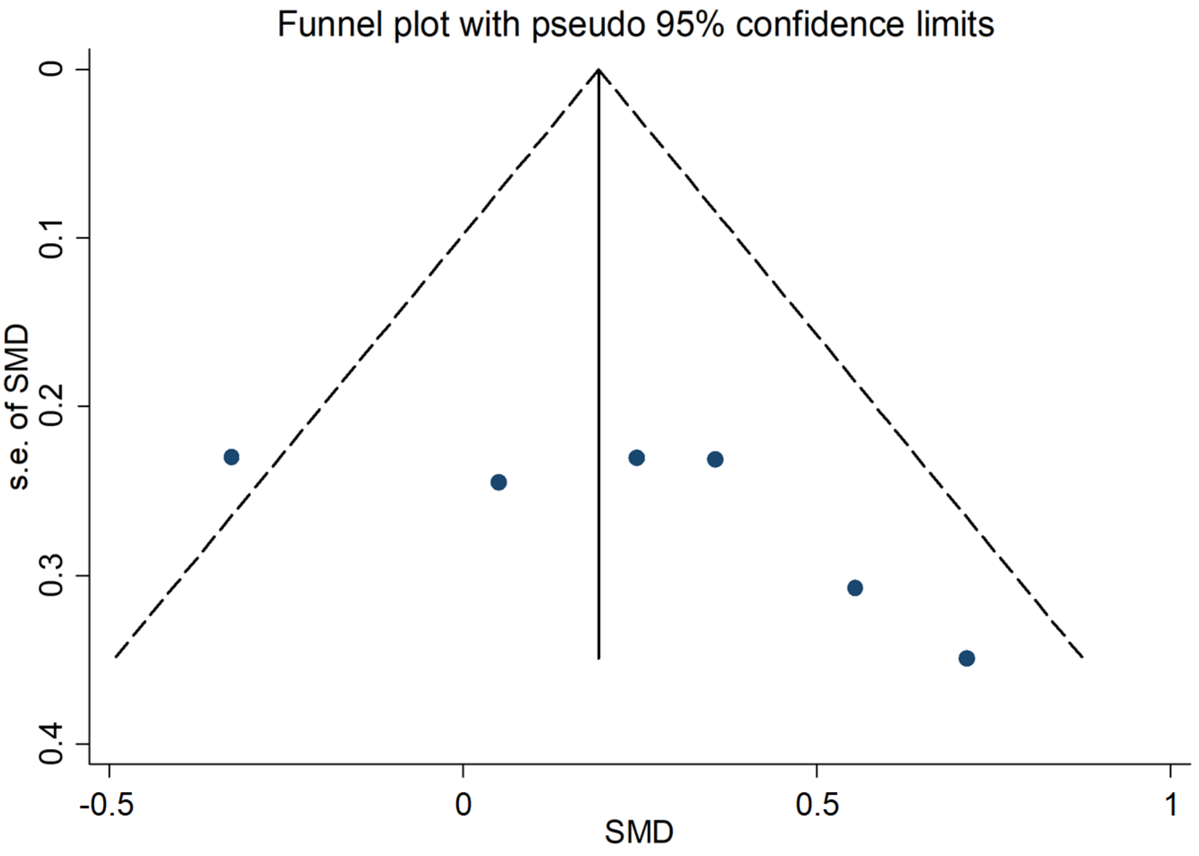

Supplementary Figure 7. Funnel plot of studies included in the cognitive flexibility meta-analysis (Trail Making Test (TMT) and The Rule Shift Cards Test / time)

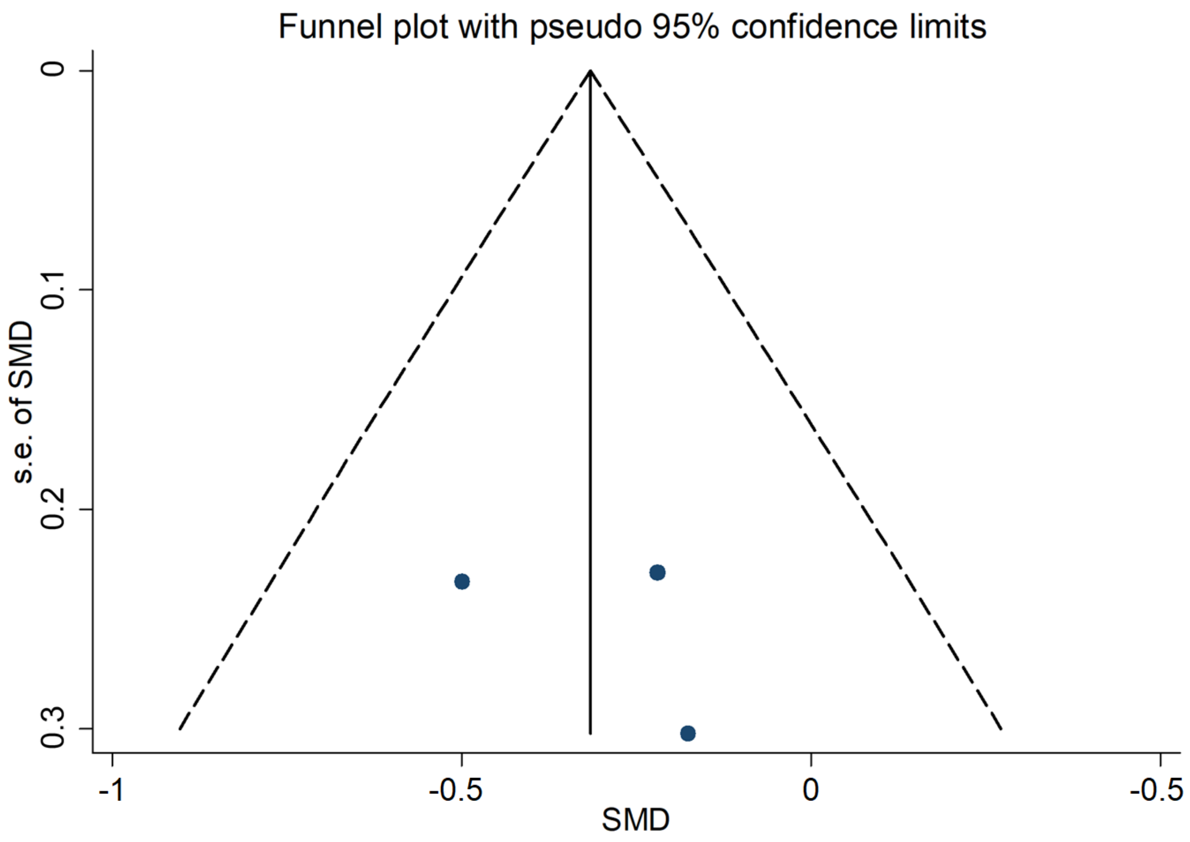

Supplementary Figure 8. Funnel plot of studies included in the working memory meta-analysis.

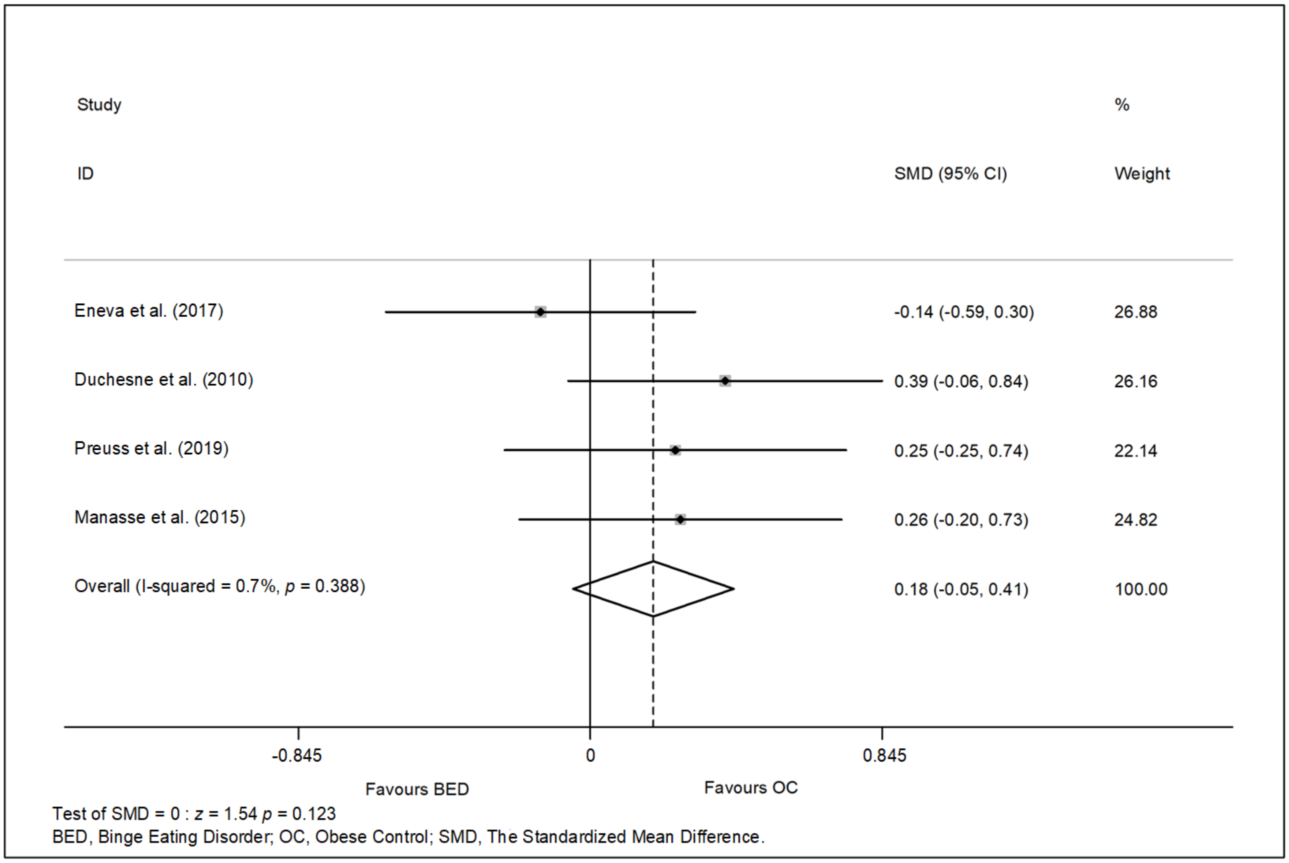

Supplementary Figure 9. Forest plot of standardized mean effect size of differences (SMD) in inhibitory control between individuals with binge eating disorder (BED) and obese healthy controls (OC) (Stroop test).

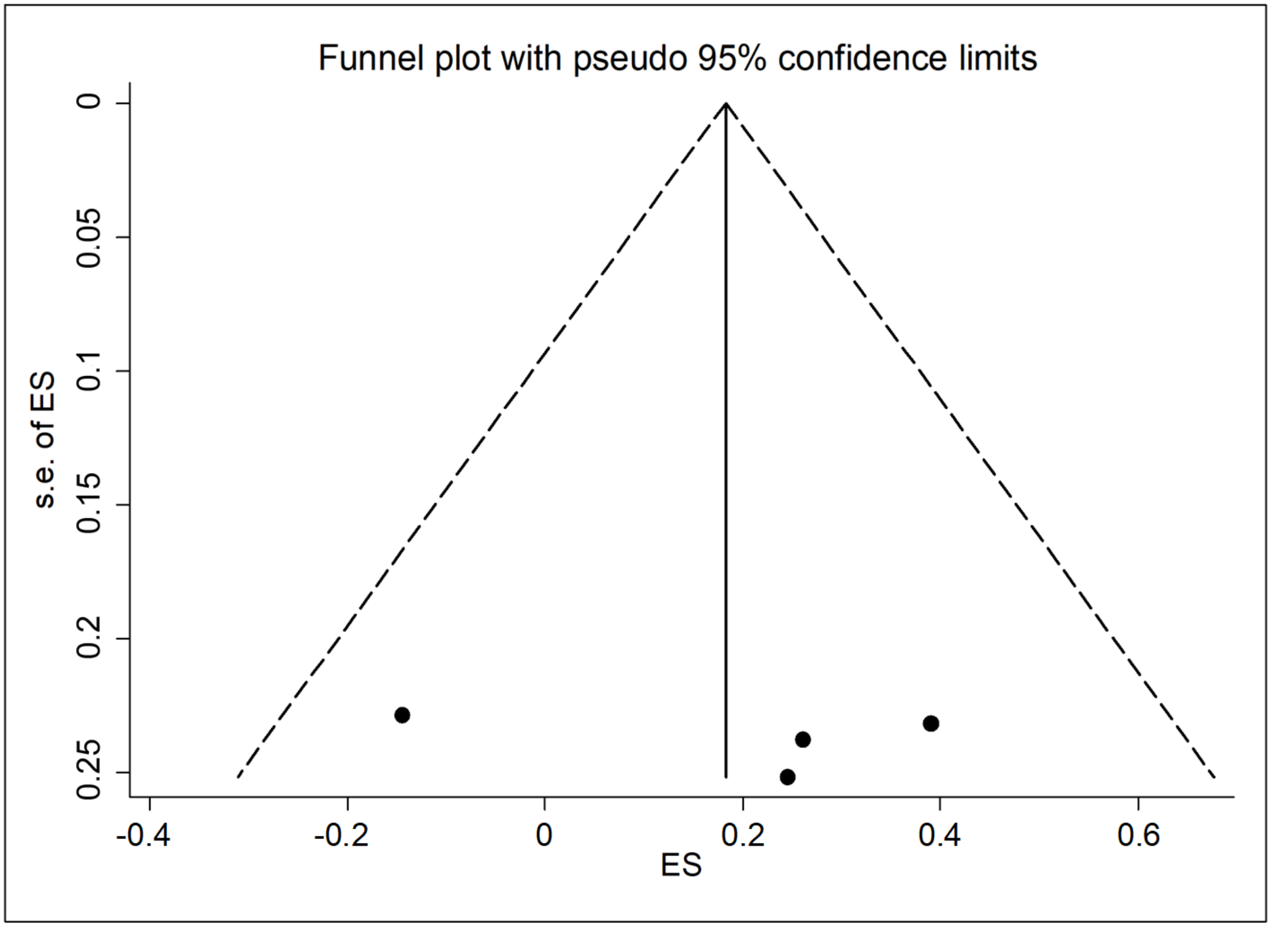

Supplementary Figure 10. Funnel plot of studies included in the inhibitory control meta-analysis (Stroop Test)

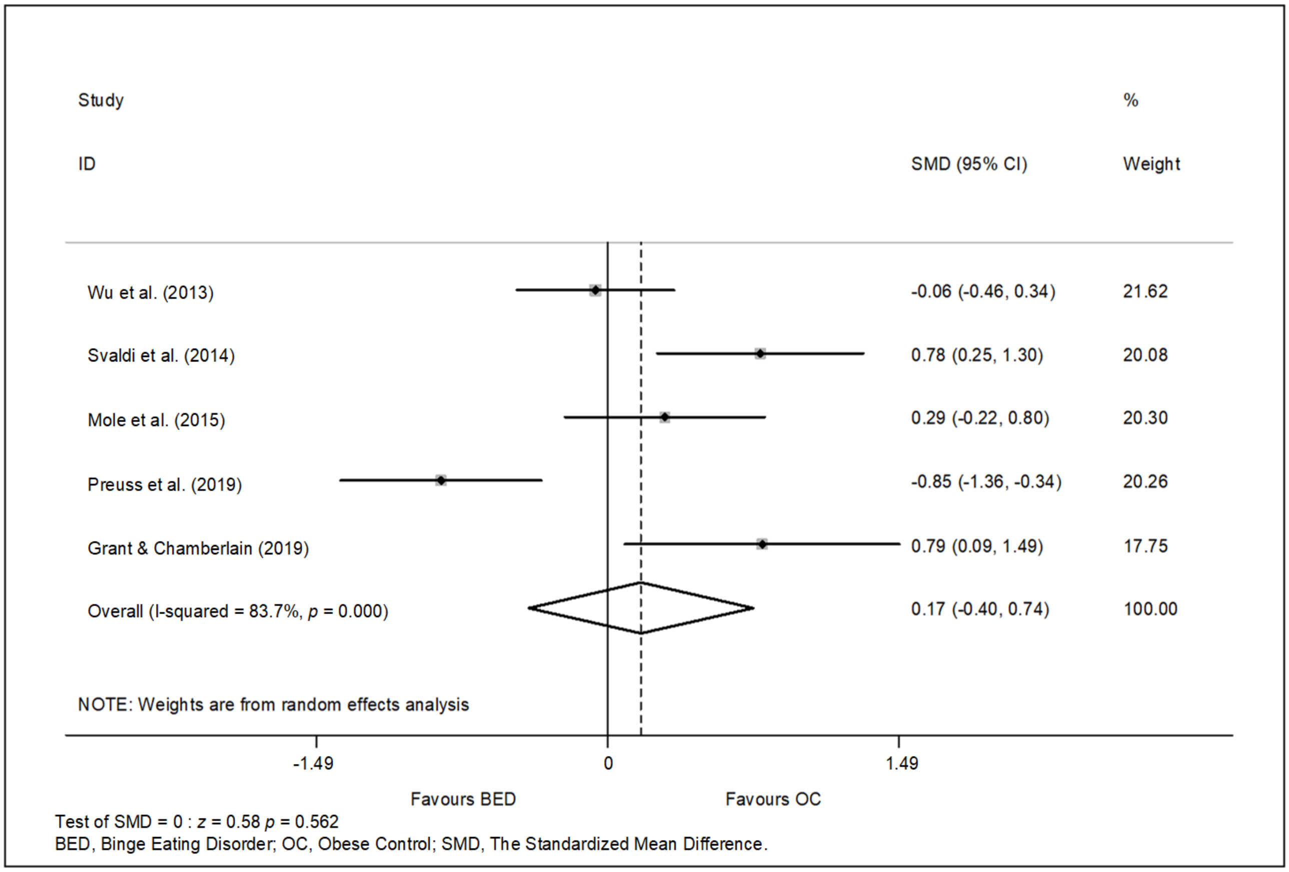

Supplementary Figure 11. Forest plot of standardized mean effect size of differences (SMD) in inhibitory control between individuals with binge eating disorder (BED) and obese healthy controls (OC). (Stop Signal Task)

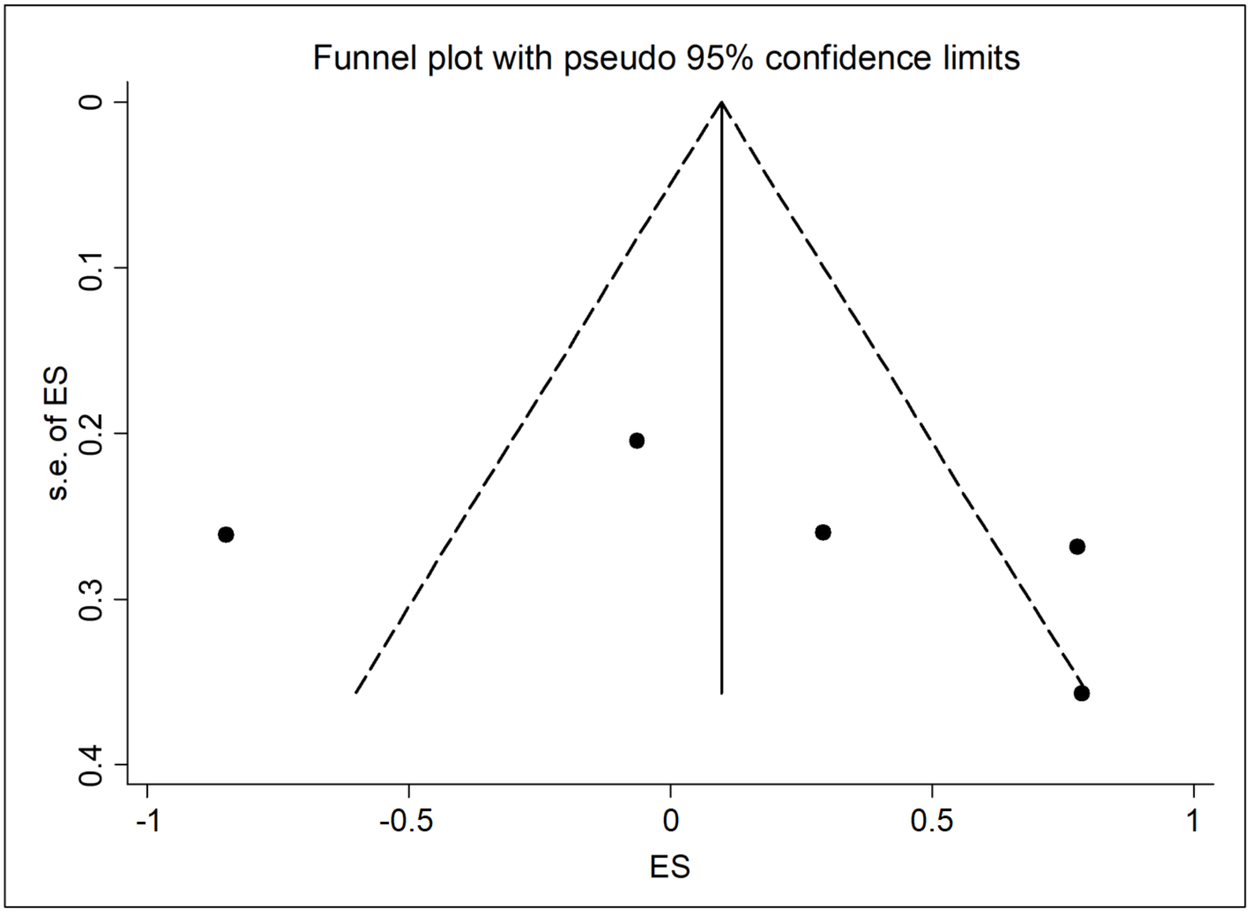

Supplementary Figure 12. Funnel plot of studies included in the inhibitory control meta-analysis (Stop Signal Task)

Author	Danner													
Year	2012													
Sample type	clinic sample													
Design	Transversal													
Female gender (%)	100%													
Age_mean_BED	38.05													
Age_sd_BED	10.97													
Age_mean_OC	44.56													
Age_sd_OC	13.36													
Age_mean_NW	36.13													
Age_sd_NW	14.09													
Years Education_mean_BED	6.21													
Years Education_sd_BED	2.59													
Years Education_mean_OC	7													
Years Education_sd_OC	2.7													
Years Education_mean_NW	6.9													
Years Education_sd_NW	2.38													
BMI_mean_BED	38.74													
BMI_sd_BED	6.25													
BMI_mean_OC	30.84													
BMI_sd_OC	3													
BMI_mean_NW	22.32													
BMI_sd_NW	1.96													
Psychiatric comorbidities	yes													
N_BED	20													
N_OC	21													
N_NW	34													
Executive Function	Decision-making													
Task	Iowa Gambling Test													
Variable	Net Score													
Mean_BED	2.74													
SD_BED	12.10													
Mean_OC	-0.94													
SD_OC	23.30													
Mean_NW	11.2													
SD_NW	18.34													

BMI, Body Mass Index; BED, Binge eating disorder; OC, Obese Control; NW, Normal Weight Control; SD, Standard Deviation.

Supplementary Figure 13. Data extraction form.

Supplementary Table 1. Characteristics of excluded studies

Reasons for exclusion	Studies
Did not include a group with a BED diagnosis	Becker KR, Plessow F, Coniglio KA, Tabri N, Franko DL, Zayas, LV, et al. Global/local processing style: explaining the relationship between trait anxiety and binge eating. *Int J Eat Disord*. (2017) 50:1264-72. doi: 10.1002/eat.22772
	Kelly NR, Bulik C. M, Mazzeo SE. Executive functioning and behavioral impulsivity of young women who binge eat. *Int J Eat Disord*. (2013) 46:127-39. doi: 10.1002/eat.22096
	Smith KE, Mason TB, Crosby RD, Engel SG, Wonderlich SA. A multimodal, naturalistic investigation of relationships between behavioral impulsivity, affect, and binge eating. *Appetite*. (2019) 136:50-7. doi: 10.1016/j.appet.2019.01.014
	Segura-Serralta M, Perpiñá C, Ciscar S, Blasco L, Espert R, Romero-Escobar C, et al. Executive functions and emotion regulation in obesity and eating disorders. *Nutr Hosp*. (2019) 36:167-72. doi: 10.20960/nh.02016
	Billingsley-Marshall RL, Basso MR, Lund BC, Hernandez ER, Johnson CL, Drevets WC, et al. Executive function in eating disorders: The role of state anxiety. *Int J Eat Disord*. (2013) 46:316-21. doi: 10.1002/eat.22086
	Harrison A, Tchanturia K, Naumann U, Treasure J. Social emotional functioning and cognitive styles in eating disorders. Br J Clin Psychol. (2012) 51:261-79. doi: 10.1111/j.2044-8260.2011.02026.x
	Allen JL, Mason TB, Stout DM, Rokke PD. Emotion specific effects on attentional bias among women with shape and weight concerns. *Cognit Ther Res*. (2018) 42:612-21. doi: 10.1007/s10608-018-9916-7
	Grant JE, Chamberlain SR. Impulsive action and impulsive choice across substance and behavioral addictions: Cause or consequence? *Addictive Behaviors*. (2014) 39:1632-9. doi: 10.1016/j.addbeh.2014.04.022
Did not evaluate an executive function examined in the review	Leehr EJ, Schag K, Dresler T, Grosse-Wentrup M, Hautzinger M, Fallgatter AJ, et al. Food specific inhibitory control under negative mood in binge-eating disorder: evidence from a multimethod approach. *Int J Eat Disord*. (2018) 51:112-23. doi: 10.1002/eat.22818
	Leehr EJ, Schag K, Brückmann C, Plewnia C, Zipfel S, Nieratschker V, et al. A Putative association of COMT Val(108/158)Met with impulsivity in binge eating disorder. *Eur Eat Disord Rev*. (2016) 24:169-73. doi: 10.1002/erv.2421
	Steward T, Mestre-Bach G, Vintró-Alcaraz C, Agüera Z, Jiménez-Murcia S, Granero R, et al. Delay discounting of reward and impulsivity in eating disorders: From anorexia nervosa to binge eating disorder. *Eur Eat Disord Rev*. (2017) 25:601-6. doi: 10.1002/erv.2543
	Manwaring JL, Green L, Myerson J, Strube MJ, Wilfley DE. Discounting of various types of rewards by women with and without binge eating disorder: Evidence for general rather than specific differences. *Psychol Rec*. (2011) 61:561-82. doi: 10.1007/bf03395777
	Bartholdy S, Rennalls S, Danby H, Jacques C, Campbell IC, Schmidt U, et al. Temporal discounting and the tendency to delay gratification across the eating disorder spectrum. *Eur Eat Disord Rev*. (2017) 25:344-50. doi: 10.1002/erv.2513
No neuropsychological assessment	Danner UN, Evers C, Sternheim L, Van Meer F, Van Elburg AA, Geerets TAM, et al. Influence of negative affect on choice behavior in individuals with binge eating pathology. *Psychiatry Res*. (2013) 207:100-6. doi: 10.1016/j.psychres.2012.10.016

	Annagur BB, Orhan O, Ozer A, Yalcin N, Tamam L. The effects of depression and impulsivity on obesity and binge eating disorder. *Bull Clin Psychopharmacol*. (2015) 25:162-70. doi: 10.5455/bcp.20130408021434
	Boeka AG, Lokken KL. Prefrontal systems involvement in binge eating. *Eat Weight Disord*. (2011) 16:e121-6. doi: 10.1007/BF03325317
	Giel KE, Speer E, Schag K, Leehr EJ, Zipfel S. Effects of a food-specific inhibition training in individuals with binge eating disorder—findings from a randomized controlled proof-of-concept study. *Eat Weight Disord*. (2017) 22:345-51. doi: 10.1007/s40519-017-0371-3
	Turton R, Nazar BP, Burgess EE, Lawrence NS, Cardi V, Treasure J, et al. To go or not to go: A proof of concept study testing food-specific inhibition training for women with eating and weight disorders. *Eur Eat Disord Rev*. (2018) 26:11-21. doi: 10.1002/erv.2566
	Alvarenga MS, Koritar P, Pisciolaro F, Mancini M, Cordás TA, Scagliusi FB. Eating attitudes of anorexia nervosa, bulimia nervosa, binge eating disorder and obesity without eating disorder female patients: Differences and similarities. *Physiol Behav*. (2014) 131:99-104. doi: 10.1016/j.physbeh.2014.04.032
	Kessler RM, Hutson PH, Herman BK, Potenza MN. (2016). The neurobiological basis of binge-eating disorder. Neurosci Biobehav Rev. (2016) 63, 223–238. doi:10.1016/ j.neubiorev.2016.01.013
Subjects had a history of BED and no current diagnosis	Lavender JM, Alosco ML, Spitznagel MB, Strain G, Devlin M, Cohen R, et al. Association between binge eating disorder and changes in cognitive functioning following bariatric surgery. *J Psychiatr Res*. (2014) 59:148-54. doi: 10.1016/j.jpsychires.2014.08.004
BED not assessed using a diagnostic tool	Córdova ME, Schiavon CC, Busnello FM, Reppold CT. Nutritional and neuropsychological profile of the executive functions on binge eating disorder in obese adults. *Nutr Hosp*. (2017) 34:1448-54. doi: 10.20960/nh.1151
	Lyu Z, Zheng P, Lu S, Qin M. Impaired conflict monitoring to food cues in women who binge eat. *Front Psychol*. (2018) 9:2585. doi: 10.3389/fpsyg.2018.02585
	Manasse SM, Espel HM, Forman EM, Ruocco AC, Juarascio AS, Butryn M, et al. The independent and interacting effects of hedonic hunger and executive function on binge eating. *Appetite*. (2015) 89:16-21. doi: 10.1016/j.appet.2015.01.013
Unable to access full text	Lee Y, Carmona NE, Shekotikhina M, Subramaniapillai M, Mansur RB, Cha DS, et al. Is binge eating a cognitive disorder? results from the international mood disorders collaborative project. *Ann Clin Psychiatry*. (2018) 30:25-31.
Inclusion of adolescents	Halevy-Yosef R, Bachar E, Shalev L, Pollak Y, Enoch-Levy A, Gur E, et al. The complexity of the interaction between binge-eating and attention. *PLoS One*. (2019) 14:e0215506. doi: 10.1371/journal.pone.0215506
No control group included	Chamberlain SR, Mogg K, Bradley BP, Koch A, Dodds CM, et al. Effects of mu opioid receptor antagonism on cognition in obese binge-eating individuals. *Psychopharmacology*. (2012) 224:501-9. doi: /10.1007/s00213-012-2778-x

Supplementary Table 2. Studies included in each executive function subdomain and in meta-analyses

Tasks	Study	Included in meta-analysis
Executive function	*Decision-making*	
Iowa Gambling Test	Davis et al. (2010)	Yes
	Danner et al. (2012)	Yes
	Aloi et al. (2018)	Yes
	Blume et al. (2018)	Yes
	Aloi et al. (2015)	No
	Dingemans et al. (2019)	No
Game of Dice Task	Svaldi et al. (2010)	Yes
	Wu et al. (2013)	Yes
Door Opening Task	Preuss et al. (2019)	Yes
Cambridge Gambling Task	Kollei et al. (2018)	No
	Grant et al. (2019)	No
	Inhibitory Control	
Stroop Test; Color-Word Interference Test	Duchesne et al. (2010)	Yes
	Manasse et al. (2015)	Yes
	Eneva et al. (2017)	Yes
	Preuss et al. (2019)	Yes
	Dingemans et al. (2019)	No
	Balodis et al. (2013)	No

Stroop match-to-sample task	Lee et al. (2017)	No
Verbal interference—word Task	Galioto et al. (2012)	No
Stop Signal Task	Wu et al. (2013)	Yes
	Svaldi et al. (2014)	Yes
	Mole et al. (2015)	Yes
	Grant et al. (2019)	Yes
	Preuss et al. (2019)	Yes
	Bartholdy et al. (2017)	No
Hayling Task	Aloi et al. (2018)	No
Mental Flexibility Task	Mobbs et al. (2011)	No
Pictorial priming paradigm	Svaldi et al. (2015)	No
Go/No Go paradigm	Hege et al. (2014)	No
	Loeber et al. (2018)	No
	Blume et al. (2018)	No
	Kollei et al. (2018)	No
	Working Memory	
Digit Span	Duchesne et al. (2010)	Yes
	Reiter et al. (2016)	Yes
	Galioto et al. (2012)	No

	Dingemans et al. (2019)	No
N-back Task with lures	Svaldi et al. (2014)	No
The Letter N-Back Task	Manasse et al. (2014)	No
NIH toolbox	Eneva et al. (2017)	Yes
	Cognitive Flexibility	
	Duchesne et al. (2010)	Yes
	Aloi et al. (2015)	No
	Reiter et al. (2017)	Yes
Trail Making Test	Eneva et al. (2017)	Yes
	Aloi et al. (2018)	Yes
	Dingemans et al. (2017)	No
	Svaldi et al. (2010)	Yes
	Duchesne et al. (2010)	Yes
	Duchesne et al. (2010)	Yes
Rule Shift Cards Test	Blume et al. (2018)	Yes
	Dingemans et al. (2017)	No
	Aloi et al. (2015)	No
	Kollei et al. (2018)	Yes
Intra/extra dimensional set shift task	Banca et al. (2016)	No
	Grant et al. (2019)	No

Pen Conditional Exclusion Task	Manasse et al. (2014)	Yes
Modified affective shifting task	Mobbs et al. (2011)	No
Switching of attention	Galioto et al. (2012)	No
	Problem-Solving	
Tower Task	Manasse et al. (2015)	No
Means-Ends Problem-Solving Procedure	Svaldi et al. (2011)	No
Action Program Test	Duchesne et al. (2010)	No
	Planning	
Zoo Map Task	Duchesne et al. (2010)	No
Tower Test	Eneva et al. (2017)	No
Maze Task	Galioto et al. (2012)	No
